# Microenvironmental effects of a non-antibiotic therapy for a chronic Polymicrobial infection Alter microbial physiology, competition, and virulence

**DOI:** 10.1093/ismejo/wraf125

**Published:** 2025-06-14

**Authors:** Cely T González, Christian Martin, Maddey Crane, Karen Gutierrez, Jacob Thomas, Lacy Remisoski, Maxwell Okros, Yousi Fu, Douglas V Guzior, Dustin Finkhouse, Christopher Bridges, Jenna Mielke, Gabriel Querido, Lienwil Padillo, Reda Girgis, Marc McClelland, Douglas Conrad, Xiaopeng Li, Robert A Quinn

**Affiliations:** Department of Biochemistry and Molecular Biology, Michigan State University, East Lansing, MI, United States; Department of Biochemistry and Molecular Biology, Michigan State University, East Lansing, MI, United States; Department of Biochemistry and Molecular Biology, Michigan State University, East Lansing, MI, United States; Department of Biochemistry and Molecular Biology, Michigan State University, East Lansing, MI, United States; Department of Biochemistry and Molecular Biology, Michigan State University, East Lansing, MI, United States; Department of Biochemistry and Molecular Biology, Michigan State University, East Lansing, MI, United States; Department of Biochemistry and Molecular Biology, Michigan State University, East Lansing, MI, United States; Department of Biochemistry and Molecular Biology, Michigan State University, East Lansing, MI, United States; Department of Biochemistry and Molecular Biology, Michigan State University, East Lansing, MI, United States; Department of Microbiology, Genetics, and Immunology, Michigan State University, East Lansing, MI, United States; Department of Biochemistry and Molecular Biology, Michigan State University, East Lansing, MI, United States; Department of Biochemistry and Molecular Biology, Michigan State University, East Lansing, MI, United States; Department of Medicine, University of California San Diego, La Jolla, CA, United States; Department of Medicine, University of California San Diego, La Jolla, CA, United States; Department of Medicine, University of California San Diego, La Jolla, CA, United States; Corewell Health, Grand Rapids, MI, United States; Corewell Health, Grand Rapids, MI, United States; Department of Medicine, University of California San Diego, La Jolla, CA, United States; Department of Pediatrics and Human Development, Michigan State University, East Lansing, MI, United States; Department of Biochemistry and Molecular Biology, Michigan State University, East Lansing, MI, United States

**Keywords:** Microbiome, Metabolome, Polymicrobial infection, Cystic fibrosis, non-antibiotic, nutrient-depletion

## Abstract

People with cystic fibrosis (pwCF) have reduced mucociliary clearance in their airways, leading to the build-up of thick, sticky mucus susceptible to opportunistic infection. A new treatment, comprised of three small molecule drugs called Elexacaftor/Tezacaftor/Ivacaftor (ETI), has improved mucociliary clearance and lung function in pwCF, but how this therapy alters lung infections is poorly understood. This study experimentally modeled the biochemical changes in airway mucus caused by ETI to determine its effect on the CF lung microbiome structure and function. We prepared Artificial Sputum Medium (ASM) with reduced primary carbon sources (amino acids, deoxyribonucleic acid DNA, and mucin) to mimic the effects of ETI on mucus biochemistry due to improved mucociliary clearance and reduced pulmonary inflammation. The control and modified ASM were inoculated with pure CF pathogens or mixed-species communities and then grown in oxic and anoxic conditions, followed by multi-omics data analysis. Although oxygen strongly altered the community structure, the nutrient depletions in ASM had little effect. Instead, the reduced carbon sources altered the physiology of the collective community and its individual pathogens. This included modified growth kinetics in addition to altered nitrogen and nucleotide metabolism. Under reduced amino acid concentrations, a known effect of ETI on the sputum metabolome, the production of both *Pseudomonas aeruginosa*’s quinolones and rhamnolipids was significantly reduced. This indirect effect of ETI translates to reduced killing of competing pathogens and reduced toxicity to epithelial cells isolated from the airways of explanted human lung tissues. These findings indicate that ETI may provide further benefit to pwCF by reducing the competition and virulence of its principal pathogen and highlight how microenvironmental effects can have powerful impacts on polymicrobial infections.

## Introduction

Cystic fibrosis (CF) is a genetic disease caused by mutations in the cystic fibrosis transmembrane conductance regulator (CFTR) gene that leads to misfolding of the CFTR protein and subsequent dysfunctional chloride and bicarbonate transport in the epithelia of multiple organs, particularly the lungs [1–3]. Disrupted ion transport leads to dehydrated mucus and compromised mucociliary clearance within the airways. Consequently, this thick sticky mucus leads to reduced host immune defense and opportunistic infection by airway pathogens [2, 4]. These pathogens can persist for decades in the lungs of people with CF (pwCF), leading to significant lung function decline, morbidity, and mortality [4]. The major CF lung bacterial pathogens, *Pseudomonas aeruginosa*, *Staphylococcus aureus,* and others, co-exist in a consortium with airway commensals, creating the complex CF lung microbiome that changes its structure and function as the disease progresses [5–8]. PwCF experience detrimental cycles of lung infection and exaggerated inflammatory responses to it, which leads to progressive lung damage, and ultimately, death. However, the CF pathophysiological landscape has changed since the recent approval of a groundbreaking CFTR modulator therapy for most pwCF (approximately 80% depending on CFTR mutations) that is composed of three small molecules known as Elexacaftor/Tezacaftor/Ivacaftor (ETI, TRIKAFTA).

ETI has significantly improved the lung function and symptoms of CF, including pulmonary infection, sweat chloride concentration, sputum production, and pulmonary exacerbations [9, 10]. The development and broad administration of ETI represents a remarkable translation of basic research into practical therapy with tangible improvements to the lives of pwCF that have continued through the 4.5 years since the initial approval by the US Food and Drug Administration (FDA) in 2019 [10–12]. Measured consequences of ETI include improvement in lung function as measured by the forced expiratory volume in 1 second (FEV_1_) (approximately 10–12%) [13], where FEV is the forced expiratory volume measure used to know how much air a person can exhale and the volume of air a person can forcibly exhale in the first second of a standardized pulmonary function test is FEV_1_. Changes in FEV_1_ are commonly used to assess disease progression and the effectiveness of therapeutic interventions, as they provide a reliable and objective measure of respiratory impairment*.* Other measured benefits of ETI include a reduction in pulmonary inflammation, sputum production, and viscosity, and a 3–4 log reduction in colony-forming units in the sputum of pathogens such as *P. aeruginosa* and *S. aureus* [10, 14, 15]. The reduction in sputum production and improved viscosity is due to better hydration of the airway mucins, which are high-molecular-weight glycopeptides that comprise the gel-like barrier of epithelial surfaces, particularly the lungs [16, 17]. Though little biochemical data is yet available, it has been reported that the concentration of amino acids and peptides in sputum is reduced on ETI [9], representing a significant shift in its chemical makeup. Invariably, the strong impacts of ETI on mucus physiology through improved CFTR function will modify and reshape the microbial niche space in the airways, but how this occurs, and its consequences on CF disease are not well understood.

The symptom improvements experienced by pwCF taking ETI since its recent approval foreshadow a bright future for those eligible for therapy (important to note that not all pwCF are eligible or can tolerate ETI) [18]. However, more recent studies have shown that despite the lower pathogen load and improved lung function, infection persists, predominantly that of *P. aeruginosa* and *S. aureus* [9–12, 16]. The persistence of infection is a troubling reminder of the prior CF era that still requires attention and novel treatment approaches in the new physiochemical mucus environment. Furthermore, the reduction in sputum production, which was the gold-standard clinical sample for microbiology, has made the study of CF lung infections more challenging. Therefore, this study conducted experiments using CF pathogens and mixed communities under conditions that mimic the known and presumed effects of ETI on the microbial niche spaces within CF mucus to better understand what the future of these infections might look like. Our primary findings show how significant changes in mucus biochemistry have less effect on community structure, but powerful impacts on community physiology, some of which may provide even further benefit to patients taking this revolutionary treatment.

## Material and methods

### Nutrient depletion experiments to mimic ETI

The principal components of Artificial Sputum Medium (ASM, originally a formula developed in [19] and then modified) [20] are amino acids, mucin, and DNA (deoxyribonucleic acid), along with other nutrients and salts. To mimic the effects of ETI improving CFTR function in CF lungs (approach described further in results), our strategy was to reduce its major carbon components separately to create “nutrient-depleted” media (ndASM) meant to represent the lung mucus environment in those on ETI. Although the current literature on the effects of ETI on mucus biochemistry and physiology is limited, we combined information from all available studies [9, 11, 12, 21] to formulate our experimental strategy. This included a separate reduction in the concentration of 1) amino acids (ASM-LowAa), previously reported and observed in [9], 2) mucin (ASM-LowMu), which is believed to represent better mucociliary clearance through CFTR potentiation and modulation, resulting in improved rheology [11, 18], and 3) DNA (ASM-LowDNA), from reduced neutrophil extracellular trap production due to lowered inflammatory signaling and neutrophil recruitment [21, 22]. Each depletion was done 10-fold from the original ASM formulation (Supplementary Table S1). For each experiment, 500 μL of the different media formulations were added into 1.50 mL Eppendorf tubes in replicates of five and inoculated with individual CF pathogens, mixed communities, or sputum. Both the ASM and ndASM samples were incubated under oxic and anoxic (85% N_2_, 5% H_2_) conditions, separately, for 48 h at 37°C, which created a fourth condition to mimic the effects of better oxygen penetration on ETI due to improved mucus clearance and rheology. To study the nutrient depletion effects collectively, a serial dilution experiment of all nutrients together was also conducted. For this experiment, four serial dilutions of ASM were prepared, including full-strength ASM, compared to 50%, 20%, 10%, or 1% concentrations of mucin, amino acids, and DNA. This serial dilution experiment was only incubated under oxic conditions.

### Synthetic bacterial community (SBC) isolation, assembly, and inoculation in ASM

Seven of the nine strains in the SBC were isolated from fresh sputum samples from pwCF, and two others were purchased from strain collections (Supplementary Table S2). Aerobic members of the SBC were initially isolated by inoculating fresh sputum in Brain Heart Infusion (BHI) broth for 24 h cultivation at 37°C. For anoxic isolation, sputum was inoculated into Brucella Broth and Chopped Meat Carbohydrate Broth (CMC broth, Anaerobe System, Inc.) and incubated anaerobically. After growth for 24 h, the cultures were streaked on different agar media for single colony isolation and identification, including Tryptic Soy Agar (TSA) supplemented with 0.5% vitamin K, Reinforced Clostridial Medium (RCM) agar [23], and Pseudomonas Isolation agar (PIA). Polymerase chain reaction (PCR) amplification of the 16S rRNA gene from each colony using the 27F and 1492R primer set, followed by Sanger sequencing to identify each isolate. To expand our community diversity, we also incorporated two strains into our SBC received from the American Type Culture Collection (ATCC), *Granulicatella elegans* ATCC 70063 and *Prevotella melaninogenica* ATCC 25845. Both the SBC mix and the single isolates were stored in glycerol 25% at −80°C. To ensure long-term viability and purity, each bacterial strain underwent weekly subculturing on selective media throughout the experiment.

To consistently assemble SBC containing a mix of nine CF isolates (details in Supplementary Table S2), bacterial cultures were first grown overnight under planktonic conditions at 37°C at 200 rpm orbital shaking. Each culture was then streaked onto individual agar plates and incubated for another 24 h at 37°C to obtain isolated colonies. From these plates, a single colony of each SBC member was inoculated with a sterile loop calibrated to 1 μL into the ASM for 24 h at 37°C to allow for initial community growth assembly (Supplementary Fig. S1). A 10 μL aliquot of this assembled culture was then used as an inoculum for each experimental culture in the nutrient depletion experiments.

### Culturing of sputum samples in ASM and ndASM

Sputum samples collected from pwCF being treated at the University of California, San Diego Adult CF clinic were mixed with glycerol (50%) and stored at −80°C for preservation (n = 7, clinical details in table S3). Collection of sputum samples was conducted under the approval of the University of California, San Diego Institutional Review Board (IRB) number #804270, with informed consent obtained from all participants. The glycerol-preserved sputum samples were subjected to nutrient depletion experiments following the same protocols as those used for the SBC, with a set of oxic and anoxic ndASM cultures for each sample. The sputum volume used was 10 μL consistently among all the experiments, and cultures were grown in oxic and anoxic conditions. Microbiome sequencing (method described below) was performed on each fresh sputum sample and its subsequent ASM culture prior to inoculation in our experiments. The data show significant differences between the initial sputum communities and the ASM experimental inoculum (Supplementary Fig. S2), which may indicate some bias in our media to enrich particular microbes in the CF airways. Additionally, to determine whether there were differences in *P. aeruginosa* metabolite production between ASM, ndASM, and actual sputum from CF airways, *P. aeruginosa* monocultures were grown in standard ASM, ndASM, and autoclave CF sputum (confirmed to be free of *P. aeruginosa),* see Supplementary Fig. S3.

### Nitrite measurements in nutrient depletion experiment

Nitrite concentration was quantified using the Griess assay [24, 25] and compared to standard curves. After growth of both the SBC and sputum communities in oxic and anoxic conditions the samples were spin down in a microcentrifuge at 14000 × *g* and 150 μL of supernatant sample was reacted with 150 μL of Griess reagent (0.1% N-(1-naphthyl)ethylenediamine-dihydrochloride, 1% sulphanilic acid, 3% H_3_PO_4_), and measured at an absorbance wavelength of 548 nm with Synergy HTX multi-mode plate reader using Gen5 software (BioTek Instruments Inc., Winooski, VT, USA) [6, 14].

### pH measurements in nutrient depletion experiment

The ASM and ndASM contain 0.04 mg/mL of phenol red dye to allow for visualization of pH changes in the media. To monitor changes in media pH during the nutrient depletion experiments, images of the tubes were captured with a Sony Cyber-Shot DSC-H300 Camera after 48 h of growth using a lightbox (example in Supplementary Fig. S4). Image analysis using Fiji was done to quantify the RBG color intensity of the media for each tube in each image to obtain a quantitative output of media pH. To transform this RGB color data to an imputed pH, ASM was formulated and buffered to a pH gradient with one pH unit increment from 5.5–8.5 and imaged identically. A standard curve of changing media pH was created from the RGB color data and then used to calculate the pH in each tube from the ASM and ndASM images. To further calibrate the data across different images, the RBG color data from a sterile control ASM in each picture was also calculated and used as a calibrant across images. These calculations do not directly measure the pH in each tube but provide an image-based estimate. Therefore, as a follow-up experiment to determine the accuracy of our image-based estimates, we directly measured the pH of sterile and inoculated cultures after 48 h and compared to our image-based pH calculation, showing good congruence (Supplementary Fig. S5).

### DNA extraction and 16S rRNA gene sequencing

Following the nutrient depletion experiments for both the SBC and sputum communities, we used 16S rRNA gene amplicon sequencing to describe the changes in the microbiome of both the SBC and the sputum communities under nutrient depletion. The bacterial DNA of SBC samples grown in ASM and ndASM were extracted following the standard protocol for biological fluids and cells described in the ZYMO Quick-DNA soil kit Soil Microbe Miniprep Kit (Cambridge Bioscience, Cambridge, UK). PCR amplification efficiency was first tested using 27F and 1492R primers that target the full-length 16S rRNA genes. For further details on the DNA extraction and PCR amplification protocols, see [26]. The PCR products were checked on an agarose gel 1% by electrophoresis to identify amplification. If amplification was achieved, the V4 hypervariable region of the 16S rRNA gene was then amplified using dual-indexed, Illumina-compatible universal primers (515F/806R) for microbiome sequencing. PCR products were batch normalized using an Invitrogen SequalPrep DNA kit. The normalization plate and the product recovered from the plates were pooled. The pool was concentrated and cleaned up using a QIAquick Spin column and AMPure XP magnetic beads. The pool was quality controlled and quantified using a combination of Qubit dsDNA HS, Agilent 4200 TapeStation HS DNA1000, and Invitrogen Collibri Illumina Library Quantification qPCR assays. This pool was loaded onto one [1] MiSeq v2 Standard flow cell, and sequencing was carried out in a 2 x 250 bp paired-end format using a MiSeq v2 500-cycle reagent cartridge for sequencing at the Michigan State University Sequencing Core. Raw sequences from both the SBC core and the sputum communities were processed using scripts from QIIME2 through the Qiita online platform (qiita.ucsd.edu [27]). Reads were denoised using Deblur after trimming sequences to a uniform length to identify amplicon sequence variants (ASVs). Taxonomic classification was performed within the Qiita platform using the SILVA reference database (version 138.1, 2020) [27]. To ensure alignment with updated taxonomy, the resulting feature table was filtered using the “filter features to Greengenes2 (GG2)” option. The sequences were rarefied at 11000 reads per sample to ensure a fair comparison of ASV counts across samples with different amplification efficiencies. The raw sequences can be accessed through the Qiita studies CF_SBC.4.23 (ID:14987, https://qiita.ucsd.edu/study/description/14987) and for pwCF samples P1-P7 (ID: 14985, https://qiita.ucsd.edu/study/description/14985). In addition, sequencing data have been deposited in the NCBI Sequence Read Archive (SRA) under BioProject accession numbers PRJNA1264509 and PRJNA1263877.

### qPCR for total bacterial load

To calculate the total bacteria load, we used the same extracted DNA and performed quantitative PCR (qPCR) of SBC total 16S rRNA genes copy number using primers 515F and 806R following the parameters used in [26, 28, 29]. Each qPCR was performed in duplicates for their respective DNA extraction using SYBR Green PCR master mix (Applied Biosystems). The products were amplified on QuantStudio3 thermocycler (Thermo Scientific). *P. aeruginosa* genomic DNA isolated from a culture with a known CFU/mL was used as a standard curve to calculate the gene copy number based on comparing C_t_ values to the ASM and ndASM samples.

### Organic extraction, liquid chromatography–tandem mass spectrometry (LC–MS/MS), and metabolomics data processing.

Organic metabolite extraction was performed by adding twice the sample volume of chilled 100% methanol, vortexing briefly, and incubating at room temperature for 2 hours. Samples were then centrifuged at 3000 × g for 10 minutes to pellet precipitated protein, and the supernatant was collected. Extracts were analyzed on a Thermo Q-Exactive Hybrid Quadrupole-Orbitrap mass spectrometer coupled to a Vanquish ultra-high-performance liquid chromatography system. Briefly, sample metabolites were separated on an Acquity C18-Reverse phase column (Waters Inc) with a 12 min chromatography run using 0.1% formic acid in acetonitrile (channel A) and Mili-Q water (channel B) gradient (98,2 to 2,98). The injection volume was 10 μL, the flow rate was 0.40 mL/min, and the column temperature was 60°C. Full MS^1^ survey scans and MS^2^ mass spectra for five precursor ions per survey scan were collected using electrospray ionization with a scan range set from *m*/*z* 100 to 1500 for the full MS mode (1–10 min of run, further mass spectrometry methodological details as described in references [9, 12]). Raw files were converted to .mzXML format through ProteoWizard and then processed with MZmine3 [30], and Global Natural Products Social Molecular Networking online platform (GNPS, https://gnps.ucsd.edu/) [31]. The resulting GNPS jobs and feature quantification tables were used for statistical and machine-learning analyses. GNPS job is available at https://gnps.ucsd.edu/ProteoSAFe/status.jsp?task=52b158ef6f3a48fb800a9bf311035c38.

### Metagenomics sequencing and analysis

To analyze the functional changes in the sputum communities (P1–P7), shotgun DNA libraries were prepared from the same extracted DNA described above using the Roche Kapa HyperPrep DNA library kit with Kapa Unique Dual-Indexed adapters following the manufacturer’s recommendations. Completed libraries were quality-controlled and quantified using a combination of Qubit dsDNA HS and Agilent 4200 TapeStation HS DNA1000 assays. The libraries were normalized to a consistent concentration, equal volumes of the normalized libraries were pooled, and the pool was quantified using the Invitrogen Collibri Quantification qPCR kit. This pool was loaded onto one lane of a NovaSeq S1 flow cell using the Xp loading kit & workflow. Sequencing was performed in a 2 x 150 bp paired-end format using a NovaSeq 6000 v1.5 300-cycle reagent kit. Base calling was done by Illumina Real Time Analysis v1.18.54 and output of RTA was demultiplexed and converted to FastQ format with Illumina Bcl2fastq v2.20.0. by the Genomics Core Research Technology Support Facility at Michigan State University (East Lansing, MI, USA). The functional annotations of the microbial metagenomic data were performed using HMP Unified Metabolic Analysis Network (HUMAnN version 3.9) within the bioBakery 3 suite, Chocophlan nucleotide database, and UniRef90 protein database [30, 31], and the MetaCyc database. Sequences were assigned organism-specific functional profiling using MetaPhlan with bowtie2 for sputum samples against human-host to remove human sequences [32, 33]. Each software was run using default parameters. Then HUMAnN’s default Reads Per Kilobase (RPK) values for gene family and pathway abundances were transformed into copies per million (CPM) units using the “humann_renorm_table” script. Finally, all the results were merged into one file through the HUMAnN_join_tables utility.

### CF pathogens competition experiments in ASM and ASM-LowAa

Co-culture experiments were done to test the relative fitness of CF pathogens in competition with each other under the normal ASM and ASM-LowAa conditions meant to mimic the effects of ETI. The experiment began with overnight cultures of *P. aeruginosa*, *S. aureus*, *Stenotrophomonas maltophilia*, and *Achromobacter xylosoxidans* [13, 33–35]. Individual colonies were inoculated with a 1 μL colony loop into BHI broth and incubated at 37°C for 24 h with constant agitation (200 rpm orbital shaking). These overnight cultures served as the inoculum for subsequent growth experiments. Monocultures were then aseptically monitored to an optical density at 600 nm (OD_600_) of 0.1 and coinoculated in a volume of 10 μL of each bacterial suspension in sets of Eppendorf tubes containing ASM and ASM-LowAa (pH 7.4 ± 0.15) in five replicates. After oxic incubation for 48 hours at 37°C, each replicated co-culture was spot-plated using serial dilutions of 0.025 mL to determine cell numbers in colony-forming units (CFU/mL) of each organism. Selective growth media, including PIA, mannitol salt agar (MSA), and PIA with 0.1% tobramycin for *P. aeruginosa*, *S. aureus*, *S. maltophilia*, and *A. xylosoxidans*, respectively, were utilized to evaluate the outcomes of competition between organisms.

### Virulence assay in lung cells

The culture supernatants from pure cultures of *P. aeruginosa* were obtained by centrifugation at 4000 × *g* for 5 min and sterilizing through a 0.45 μm filter (polyethersulfone membrane, Fisher Scientific) to remove the microbial cells, transferred to an Eppendorf tube and frozen at −80°C until use in the virulence test. Here, we developed protocols to culture both large and small airway epithelial cells at the air-liquid interface (ALI) using transbronchial (small airway) and endobronchial (large airway) biopsies obtained during routine clinical bronchoscopies from lung transplant recipients. These donor tissues were free of acute or chronic rejection, ensuring the biopsies represent healthy airway epithelium. Cultures were utilized once the epithelial cells reached a well-differentiated state, as described previously [36]. After written informed consent (IRB#: 2020–188 from the Corewell Health group). Small airway epithelial cells successfully formed well-differentiated monolayers and expressed region-specific markers, including SCGB3A2, a secretory protein highly expressed in small airways. We confirmed the presence of regionally distinct gene expression profiles, such as ATP12A in large airways and SCGB3A2 in small airways [37, 38]. Transbronchial biopsies were taken from a lower lobe for small airway cells, and endobronchial biopsies were taken from the 3rd bronchial bifurcation for large airway cells. Samples were placed immediately in culture media on ice and transported to the laboratory. Airway epithelial cells were expanded using established methods developed to conditionally re-program airway epithelial cells [36]. We use expanded cells in passage 1 to ensure those cells maintain native properties. Large and small airway cells from the same donor (n = 5) were challenged with supernatants of *P. aeruginosa* culture for 6 hours, and a Ussing chamber assay was used to measure the trans-epithelial electrical resistance (TEER) or cell barrier integrity, which is widely used to evaluate epithelial barrier function by quantifying the integrity of tight junctions in cellular monolayers [39]. TEER measures electrical resistance across epithelial cell layers, reflecting the tightness of intercellular junctions [40]. Higher resistance (expressed in Ohm·cm^2^) indicates stronger barrier integrity, while lower values suggest permeability or junction disruption [41].

### Statistical analysis

To assess microbiome alpha diversity the Shannon index was computed for both datasets (SBC and pwCF P1-P7) using the diversity function in vegan version 2.6–4 [42]. Further alpha-diversity metrics including the Simpson and Fisher indices were calculated for the serially diluted media experiment. Beta-diversity was computed between the ndASM and ASM communities using the Weighted UniFrac distance to quantify dissimilarities between sample pairs. We performed PERMANOVA (adonis test) using the adonis2 function from the vegan (v2.6.4) R package. Changes in diversity, nitrite concentration, pH, total bacterial load, and pathway abundances were assessed using the Kruskal–Wallis tests. Post-hoc comparisons were conducted using Dunn’s test with corrections for multiple comparisons. Random forests (RF) analysis was employed to classify and regress the microbiome and metabolome data by ndASM condition or serial dilution, respectively, with variable importance plots to identifying the primary variable driving differences between conditions. Mann–Whitney U-tests were then used for pairwise significance testing between the ASM control media to ndASM conditions pairwise. For comparisons between two groups in the co-culture assay, Student’s t-tests were employed. Finally, testing of changes in TEER were tested in control media or inoculated with *P. aeruginosa* using a dependent t-test. Statistical significance was defined as a *P* value ≤0.05 and represented as ^*^: *P* < 0.05; ^**^: *P* < 0.01; ^***^: *P* < 0.001.

## Results

### Strategy to mimic the effects of ETI using modified artificial sputum medium (ASM)

**Figure 1:**
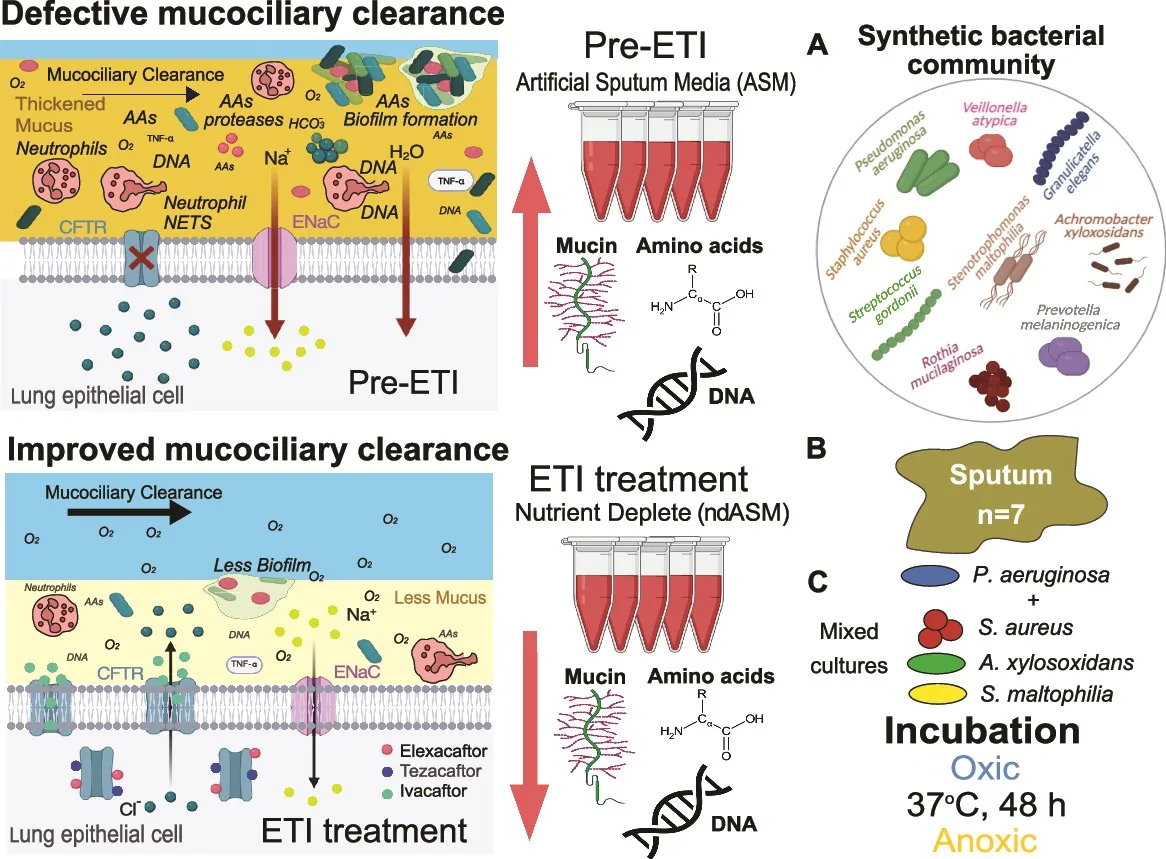
Schematic of our approach to mimic ETI treatment effects on mucus biochemistry and physiology. Synthesizing findings from previous studies and drawing on knowledge of CFTR dysfunction, we created nutrient-depleted ASM (ndASM) by reducing amino acids (ASM-LowAa), mucin (ASM-LowMu), and DNA (ASM-LowDNA) concentrations 10-fold. ETI treatment is expected to create a thinner mucus layer with less mucus overall, greater oxygen penetration, and a reduced concentration of carbon sources, such as amino acids and DNA, which are sourced from the action of neutrophil proteolysis and extracellular traps, respectively. The experiments were conducted in replicates (n = 5) under oxic and anoxic conditions, including a synthetic bacterial community (SBC) of nine lung microbiome isolates, sputum samples collected from pwCF, and individual species in competition experiments. Figure partially created with BioRender.com.

Drawing on knowledge of how CFTR dysfunction alters mucus properties and synthesizing existing research on the impact of ETI on mucus biochemistry and physiology [9, 11, 12, 21], we developed an approach to simulate the effects of ETI treatment by creating a modified ASM for bacterial culturing. Our principal strategy was to dilute key carbon sources separately, including amino acids, mucin, and DNA and then compare to dilution of all three together. The resulting nutrient-depleted media (ndASM) formulations are denoted as ASM-LowAa, ASM-LowDNA, and ASM-LowMu (Supplementary Table S1, Fig. 1). These different media formulations were then inoculated with microbial samples including pure cultures, mixed communities, and sputum followed by multi-omics analysis. As a fourth mechanism of mimicking the effects of ETI, all cultures were incubated both aerobically and anaerobically, with the former meant to represent the ETI condition due to better oxygen penetration into mucus (Fig. 1).

### Changes in community composition, nitrite consumption, and pH of SBC caused by ASM nutrient depletions

**Figure 2:**
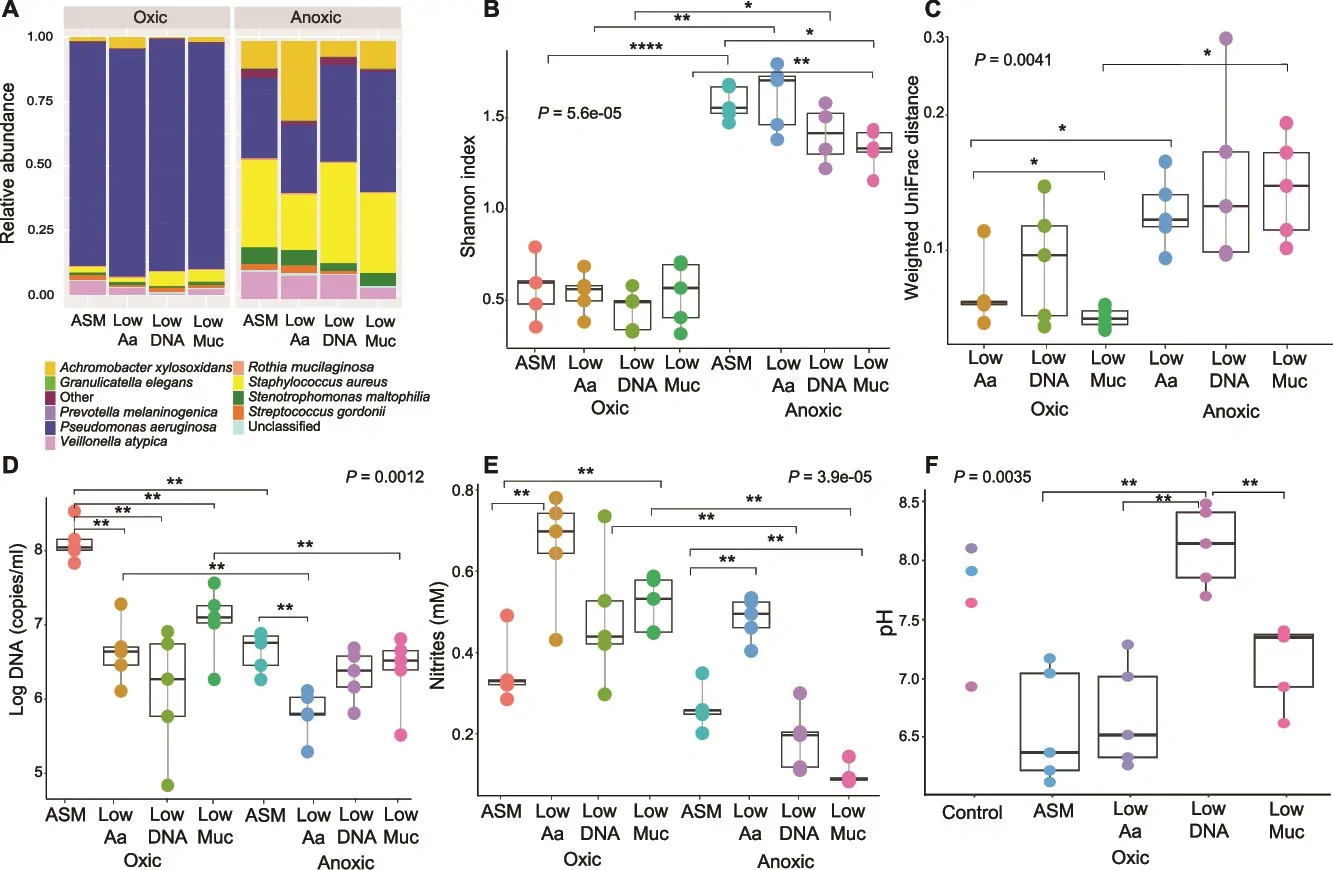
SBC changes in single-nutrient depletion conditions. (A) the 16S rRNA gene microbiome profile of the SBC after growth in nutrient-depleted conditions, the bar plots represent the mean of each ASV from the five replicates. (B) Alpha diversity was calculated with the Shannon index for the various ndASM conditions grown aerobically and anaerobically. (C) Beta-diversity was calculated with the weighted UniFrac distance between each ndASM and the control ASM. (D) Total bacterial load (log_10_ 16S rRNA gene copy number/mL) across the various media conditions. (E) Nitrite concentration in the various media after 48 hours of growth of the SBC. Statistical tests were performed using a Kruskal-Wallis test for the entire data set and then post-hoc Dunn’s test, ^**^P < 0.01, ^*^P < 0.05. f) Boxplots of the SBC pH changes in ASM and ndASM based on image analysis. pH of sterile controls for each condition is shown as single points with any replicates averaged.

The SBC comprising 9 strains representative of the CF sputum microbiome was initially cultured in ASM and ndASM conditions with each nutrient depleted separately and incubated either aerobically or anaerobically. The strongest effect on SBC structure came from growth under different oxygen conditions. Analyzing the community taxonomic profile showed that the oxic condition resulted in a dominance of *P. aeruginosa* regardless of the nutrient depletions with higher relative abundances of *A. xylosoxidans*, *S. aureus,* and *Veillonella atypica* observed under anoxic conditions (Fig. 2A). The communities were also more diverse and even in an anoxic environment, with particularly strong effects from the Shannon and Simpson indices which account for community evenness (Fig. 2A, B, Supplementary Fig. S6, *P* < 0.001, Kruskal-Wallis, Fig. 2B). Within the anoxic cultures, a lower mucin concentration (ASM-LowMu) decreased alpha diversity compared to control ASM (Dunn’s test *P* = 0.034, Fig. 2B). Comparing beta diversity by calculating the weighted UniFrac distance between each ndASM condition and the ASM control showed that the nutrient depletions had a larger effect on the community structure when grown anaerobically with little community variation in the SBC when grown aerobically (Fig. 2C). As anticipated, due to lowered carbon availability, a reduction of the total bacterial load was observed in the ndASM conditions, particularly evident in oxic conditions (Kruskal-Wallis *P* < 0.01, see Fig. 2D). Similarly, under anoxic conditions the ASM control cultures reached the highest total density, but this was only significant when compared to ASM-LowAa. There were no specific microbial ASVs statistically significant between the nutrient depletions and control ASM cultures within oxygen conditions. Collectively, the nutrient depletion experiments had a small effect on community structure, except for the reduction in the total bacterial load and relative abundance of *P. aeruginosa* in the anoxic environment compared to oxic conditions.

Due to the lower total bacterial load under anoxic conditions, we were curious whether there was evidence of sufficient anaerobic respiration with nitrite as an electron acceptor to fuel community growth, specifically that from *P. aeruginosa*. Prior to community inoculation in the ASM, the nitrite concentration was 1.27 μM ± 0.06, but after growth, this concentration was significantly reduced, indicating nitrite respiration by the SBC (Fig. 2E). The bacteria consumed less nitrite in ASM-LowAa both aerobically and anaerobically. This observation suggests a shift in bacterial nitrogen metabolism from amino acid depletion, possibly due to the overall lower nitrogen input in the ASM-LowAa condition (Dunn’s test *P* < 0.05, Fig. 2E) [6, 43, 44]. This reduction in denitrification may have implications for limiting anoxic growth strategies of CF pathogens and suggests how the microbiome may be impacted by treatment, highlighting changes in microbial interactions and adaptation that could influence disease progression in the airway mucus of those on ETI.

Because the ASM contains a phenol red pH dye, changes in pH can be visually observed in the SBC cultures and calculated using buffered standards (see methods and supplemental material). The nutrient depletions in the SBC had a significant effect on culture pH in the oxic condition, with the ASM pH dropping compared to a sterile control (mean = 6.59 +/− 0.48), except for the ASM-LowDNA condition, where the pH of the culture significantly increased to a mean of 8.11 (+/− 0.34) (Tukey HSD, *P* < 0.01, Fig. 2F). Collectively, changes in nitrate respiration, pH, and total bacterial load indicated that although the community structure was consistent under nutrient-depleted conditions, community function and total growth varied.

**Figure 3:**
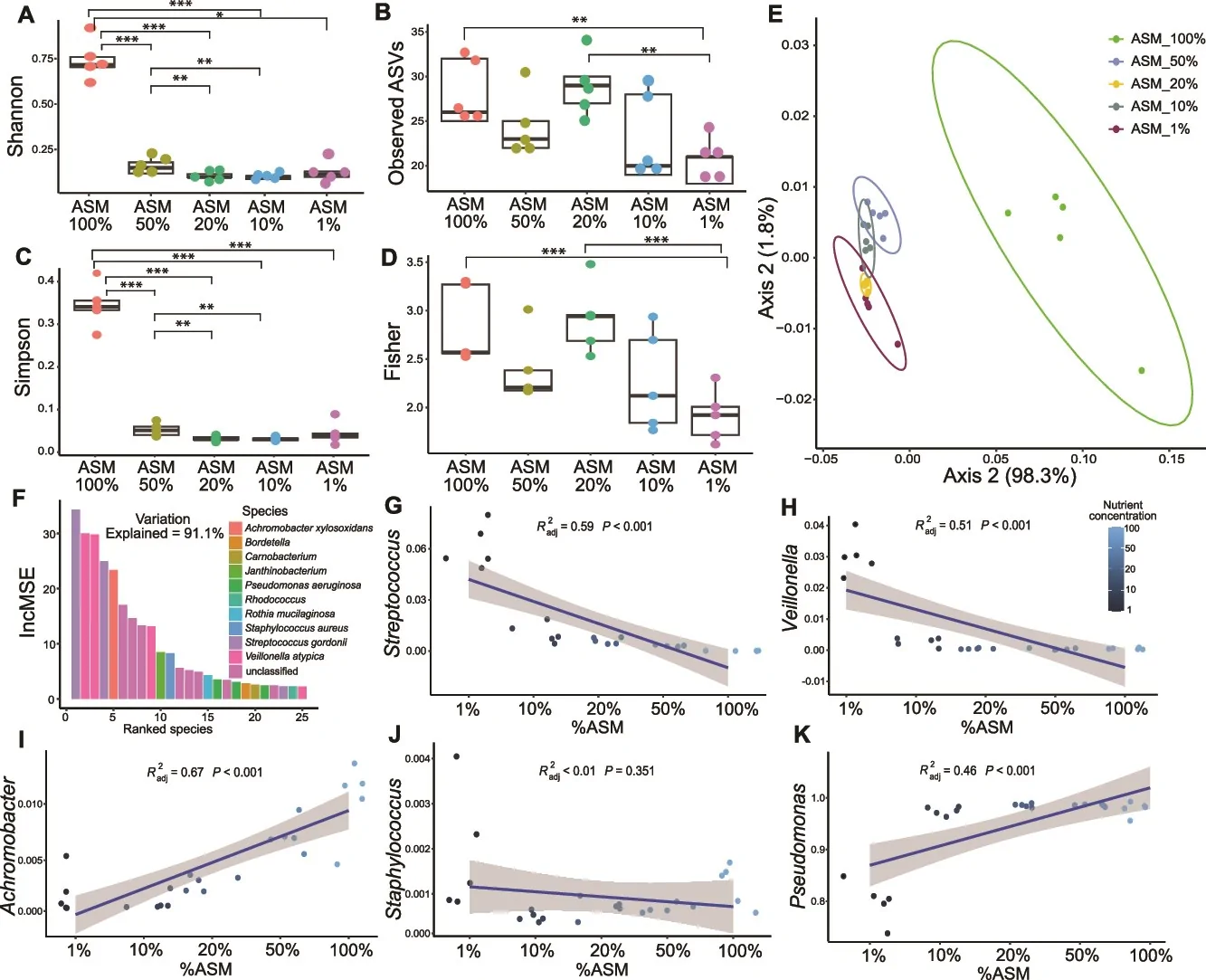
Alpha diversity, beta diversity, and microbial taxonomic changes of SBC across different ASM serial nutrient dilutions. (A–D) boxplots showing alpha diversity metrics across artificial sputum media (ASM) dilutions. E) Weighted UniFrac principal coordinate analysis (PCoA) illustrating beta diversity of SBC across ASM dilutions. Clustering patterns demonstrate the impact of nutrient availability on community structure, with the axes representing the percentage of explained variance. PERMANOVA tests on the ASM depletions statisics: *R*^2^ = 0.944101, *P* < 0.001, *F* = 84.447. (F) Feature importance plot from random Forest regression analysis showing the explanatory power of key bacterial species for the observed variations in community composition (IncMSE = increase in mean squared error). (G–K) linear regression plots depict relationships between ASM serial dilutions and the relative abundances of specific bacterial taxa: *Streptococcus, Veillonella, Achromobacter, pseudomonas*, and *staphylococcus*. Regression equations, adjusted *R*^2^ values, and *P* values are indicated for each taxon. Shaded regions represent 95% confidence intervals. Significant differences were determined using pairwise statistical comparisons (^*^P < 0.05, ^**^P < 0.01, ^***^P < 0.001).

To test the combined effects of the three primary nutrient depletions, a follow-up experiment was performed with the SBC and a *Pseudomonas*-containing sputum community where all three nutrients were serially diluted together to determine the effect of loss of all major carbon sources on the CF microbiome, which may be mostly relevant to changes observed from ETI treatment. In contrast to diluting a single nutrient at a time, this experiment showed a significant effect on the community structure, particularly the SBC diversity (Fig. 3A-F). At only a 50% concentration of the main carbon sources, the Shannon and Simpson indices drop significantly, with little change from further dilution. A similar trend is observed in the sputum community P1 (Supplementary Fig. S7A-F). Measures of community richness, including the abundance-based coverage estimator (ACE diversity) and observed ASVs, showed a more progressive reduction with serial dilution where microbes were lost at each dilution step. This effect was observed in the beta-diversity analysis as a large shift in community structure in the first dilution and progressive, more subtle changes thereafter. The analysis, based on Weighted UniFrac distances, confirmed a significant impact of the experimental condition on community structure (*R*^2^ = 0.944, *P* < 0.001, *F* = 84.447) (Fig. 3G). Similarly, for the sputum community, beta diversity analysis using Weighted UniFrac distances demonstrated a clear effect of serial dilutions on community composition (Supplementary Fig. S7G). Random forests regression analysis on the dilution factor prioritized the organisms that responded the strongest and identified *Streptococcus* and *Veillonella* as significantly reduced from nutrient depletion, whereas *Achromobacter* and *Pseudomonas* increased their relative abundances as nutrients were depleted (Fig. 3I-M). Whereas the sputum community showed a similar increase in *P. aeruginosa*, other key bacteria, such as *Veillonella*, exhibited distinct patterns of change (Supplementary Fig. S7I-L). This experiment shows that nutrient depletions mimicking ETI can affect community structure in our microcosm experiments but only when the total carbon load is reduced collectively.

### Nutrient depletions alter sputum community physiology

**Figure 4:**
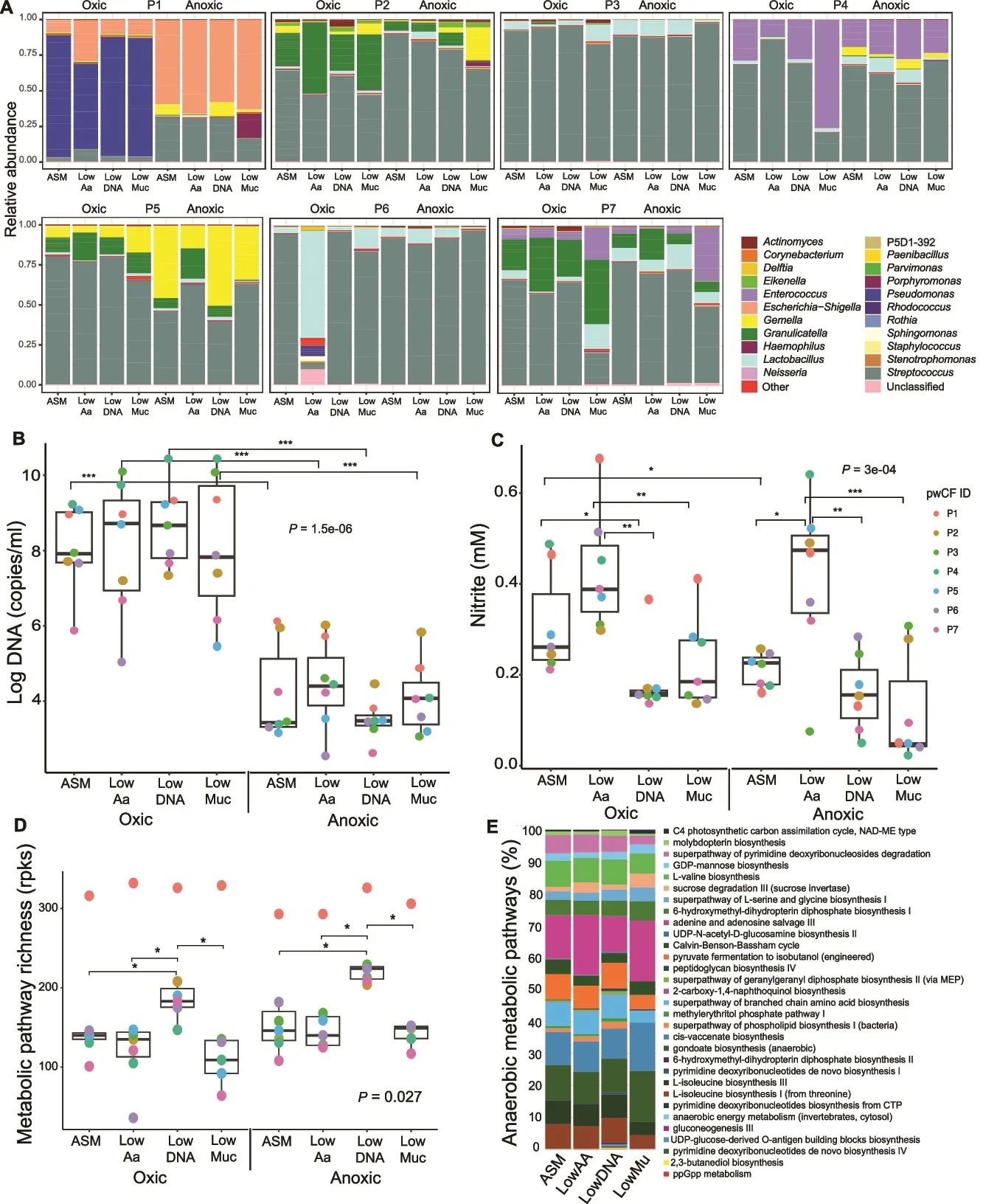
The impact of nutrient depletion on fresh-sputum microbiome communities from pwCF using multi-omics analysis to identify significant changes under this nutrient stress. (A) the 16S rRNA gene microbiome composition of sputum communities from seven pwCF under nutrient-depleted conditions. (B) Total bacterial load as calculated using qPCR quantification of the 16S rRNA gene copies per mL of media. (C) Nitrite concentration after growth of the seven sputum samples under ASM and ndASM conditions (Kruskal-Wallis test, *P* < 0.001). (D) the richness of metabolic pathways present in the pwCF samples was analyzed using shotgun metagenomics. (E) Relative abundance of the top 30 metabolic pathways from the RF classification of the cultured sputum communities under oxic conditions. Statistical tests are first performed across treatments with the Kruskal-Wallis, followed by the post-hoc Dunn’s test (^***^P < 0.001, ^**^P < 0.01, ^*^P < 0.05).

After finding subtle changes in SBC structure under single nutrient-depleted conditions but indications of altered community physiology, we explored this potential effect of ETI further by culturing sputum communities from seven pwCF with the same experimental design. Here, we used 16S rRNA gene sequencing, shotgun metagenomics, and metabolomics to better characterize community structural and functional changes. The sputum communities were mostly distinct based on patient source, with only P1 containing detectable amounts of *P. aeruginosa* after culture in ASM, and the others (P2-P7) dominated by *Streptococcus* (Fig. 4A, Supplementary Fig. S2). *P. aeruginosa* was detected in the clinical culture history from 4 of the 7 sputum inocula but was only detected in the ASM community from P1. The community structures showed some changes in ndASM compared to the full ASM, but these were highly variable, and the collective alpha and beta diversity across the seven patients were not significantly different by nutrient depletion compared to control ASM (Kruskal-Wallis *P* = 0.29, Supplementary Fig. S8A-B). The patient harboring *Pseudomonas* had differences in community structure under oxic or anoxic conditions, with reduced *Pseudomonas* relative abundances under the latter. *G. elegans* showed a similar phenomenon in anoxic conditions but had only small differences in the various ndASM cultures. There were some other community composition changes, particularly in the ASM-LowMu samples from P2, P4, and P7, but these were not consistent across the different subjects and were subtle overall. Like the SBC results, the total bacterial load was lower under anoxic conditions, though in this experiment there was evidence that some communities struggled to grow at all, reaching less than 10^4^ 16S rRNA gene copies/mL anaerobically (Fig. 4B). Again, like the SBC results, there were alterations in the concentrations of nitrite between the ASM and ndASM conditions, with ASM-LowAa having higher nitrite concentrations across the seven subjects after growth in both oxic and anoxic conditions (Dunn’s test *P* < 0.001, Fig. 4C). There was higher variability in pH from control media in the sputum cultures with some subjects having culture pH ranging from 5 to 8 (Supplementary Fig. S8C). Despite this variability, the pH of ASM-LowMu cultures was significantly higher than the others (Supplementary Fig. S8C).

As a means of further exploring the physiological changes under ETI-mimicking conditions, we performed shotgun metagenomics on the extracted DNA from these sputum cultures to analyze changes in community metabolic pathways. The sputum sample with *Pseudomonas* (P1) consistently exhibited a greater number of metabolic pathways compared to all other organisms, likely due to the large and flexible genome of *P. aeruginosa*, enabling it to perform a broader range of metabolic activities. When comparing ASM to ndASM there was an increased metabolic pathway richness in ASM-LowDNA cultures that was statistically significant compared to ASM both aerobically and anaerobically (Kruskal-Wallis, post hoc Dunn’s test *P* < 0.05, Fig. 4D). Deeper analysis of the metagenome pathways using RF a classification by media type for the oxic cultures further identified the ASM-LowDNA as having a unique metagenome across the subjects (Supplementary Table S4). The top 30 metabolic pathways from the RF variable importance plot included a number related to nucleotide synthesis and amino acid synthesis. Plotting the abundance of these pathways by media type showed that the nucleotide synthesis pathways were more abundant in DNA-depleted media, which would be expected as the microorganism may be starving for nucleotides in a DNA-depleted environment, microorganism must rely on de novo nucleotide synthesis to meet their cellular requirements. Lacking exogenous DNA as a nucleotide source would upregulate or rely more heavily on their own nucleotide biosynthesis pathways. But other pathways were elevated in ASM-LowDNA as well, including 2–3 butanediol synthesis and peptidoglycan synthesis (Fig. 4E, Supplementary Fig. S9). Collectively, this metagenome analysis of sputum samples cultured in ndASM further supports the notion that nutrient depletions have less effect on community structure but are altering community metabolic pathways (Supplementary Fig. S10).

### Nutrient-depleted conditions modify *P. Aeruginosa* virulence metabolite production

To further investigate the impact of nutrient depletion on community physiology, we employed LC–MS/MS to profile metabolite alterations in SBC and sputum samples grown in ASM and ndASM conditions. The nutrient depletions, particularly amino acids and DNA depletions, were expected to influence the metabolome due to our LC–MS/MS methods directly measuring some of the diluted media components. Therefore, we focused on metabolites and pathways altered by the nutrient depletions outside of the actual media components.

**Figure 5:**
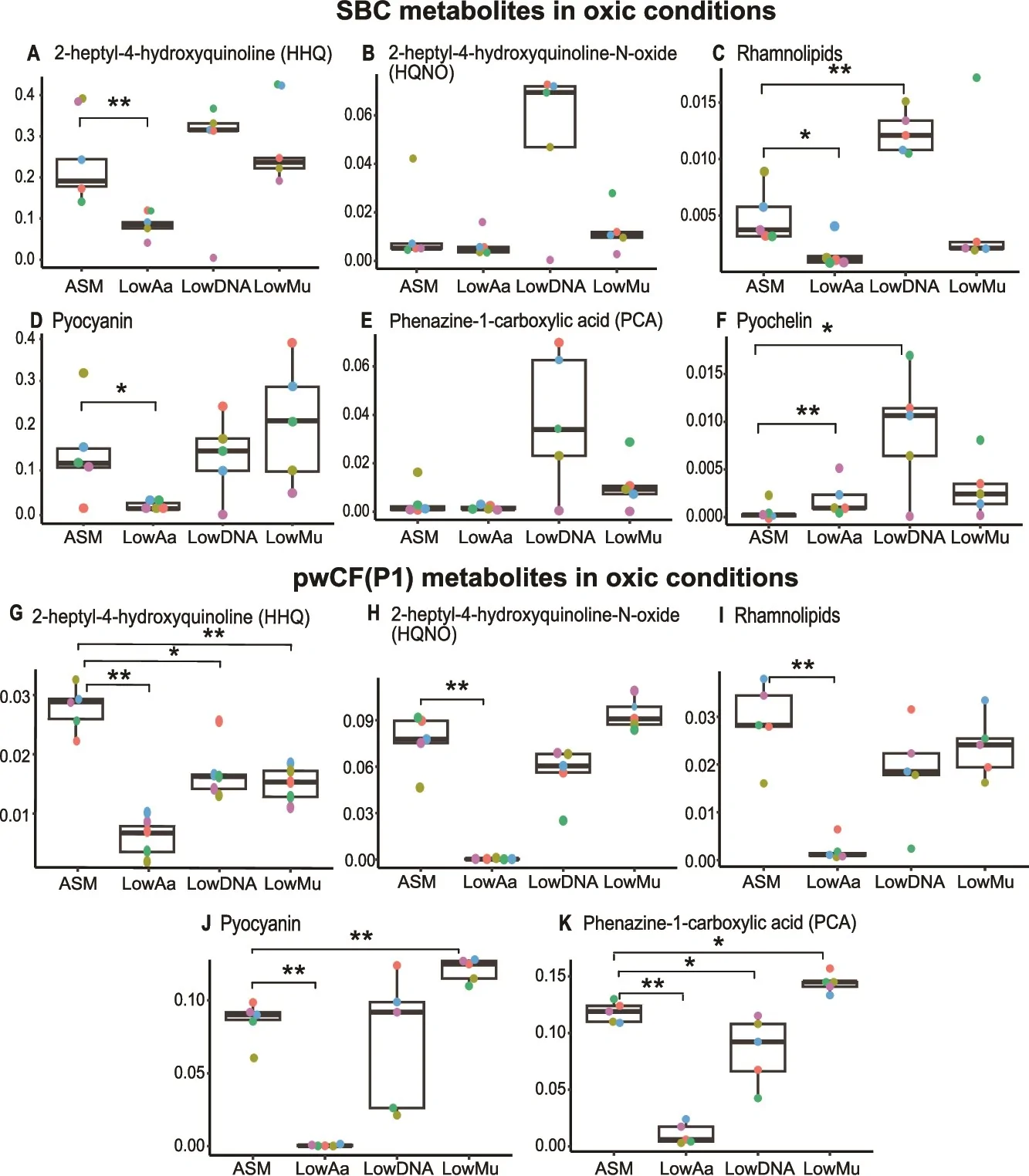
*P. Aeruginosa* metabolite changes in SBC and sputum cultures under nutrient depletion incubated aerobically. Each metabolite is plotted as its normalized abundance to the whole metabolome and each point represents an experimental replicate. (A) HHQ (2-heptyl-4-hydroxyquinoline) levels, (B) HQNO (2-heptyl-3-hydroxy-4(1H)-quinolone N-oxide) levels, (C) Rhamnolipids levels, (D) Pyocyanin levels, (E) PCA (Phenazine-1-carboxylic acid) levels, (F) Pyochelin levels. SBC cultures under oxic conditions, and G-K) sputum cultures from the sputum of subject P1. Mann–Whitney to media comparison, ^**^P < 0.01, ^*^P < 0.05.

An RF machine learning algorithm was used to classify the SBC metabolomes by oxic and anoxic culture and across the nutrient depletions. The RF classification by oxygen condition achieved a 0% error rate, highlighting the significant impact of the oxygen environment on community metabolism (Table S6). Variable importance plots identified that *P. aeruginosa* virulence metabolites were among the top classifiers of oxygen conditions, representing 7 out of the 10 strongest annotated molecules (Table S7). Similarly, RF classification strongly differentiated each ndASM culture from the ASM control (Table S8). As anticipated, many of the strongest classifying metabolites were those reduced in the media (e.g., phenylalanine for ASM-LowAa, Table S9). *P. aeruginosa* specialized metabolites, including quinolones and pyochelin, were also prominent classifiers of nutrient depletions under oxic and anoxic conditions and were therefore explored further. The ASM-LowAa and ASM-LowDNA had contrasting effects on the abundance of *P. aeruginosa* virulence metabolites from the SBC cultures. The ASM-LowAa culture condition, meant to mimic the reported reduction of amino acid concentrations in sputum of pwCF on ETI, resulted in lowered 2-heptyl-4-hydroxyquinoline (HHQ), pyocyanin and total rhamnolipids than then control ASM (Mann–Whitney U-test, *P* < 0.05 Fig. 5). Under the ASM-LowDNA condition the total amount of rhamnolipids was increased compared to the ASM control. Overall, these *P. aeruginosa* metabolites were far more abundant in oxic conditions (approximately 100-fold higher). Therefore, we further analyzed changes under this culture condition in subject P1, which produced a sputum sample with significant amounts of *P. aeruginosa,* providing a similar experiment to the SBC design.

Consistent with SBC findings, nutrient depletions for P1 sputum affected *P. aeruginosa* specialized metabolite production, including reduced quinolones, rhamnolipids, pyocyanin, and phenazine-1-carboxylic acid (PCA) under ASM-LowAa, but the effects of ASM-LowDNA were less evident (Mann–Whitney U-test, *P* < 0.05, Fig. 5G-K). The *P. aeruginosa* community behavior in the sputum experiment did not produce detectable pyochelin (Supplementary Fig. S11), possibly due to strain level differences in pyochelin production or manipulation of the molecule by other species in the community [45]. Finally, to further demonstrate that the nutrient depletions altered *P. aeruginosa* virulence metabolite production, we cultured the organism alone in ndASM and found similar changes, particularly reduced production of quinolones and rhamnolipids compared to a control media. These same metabolites were detected when the bacterium was cultured in autoclaved sputum as a media, validating their production in sputum as well (Supplementary Fig. S3).

### Nutrient-deplete conditions reduce *P. Aeruginosa* virulence factor production and fitness

**Figure 6:**
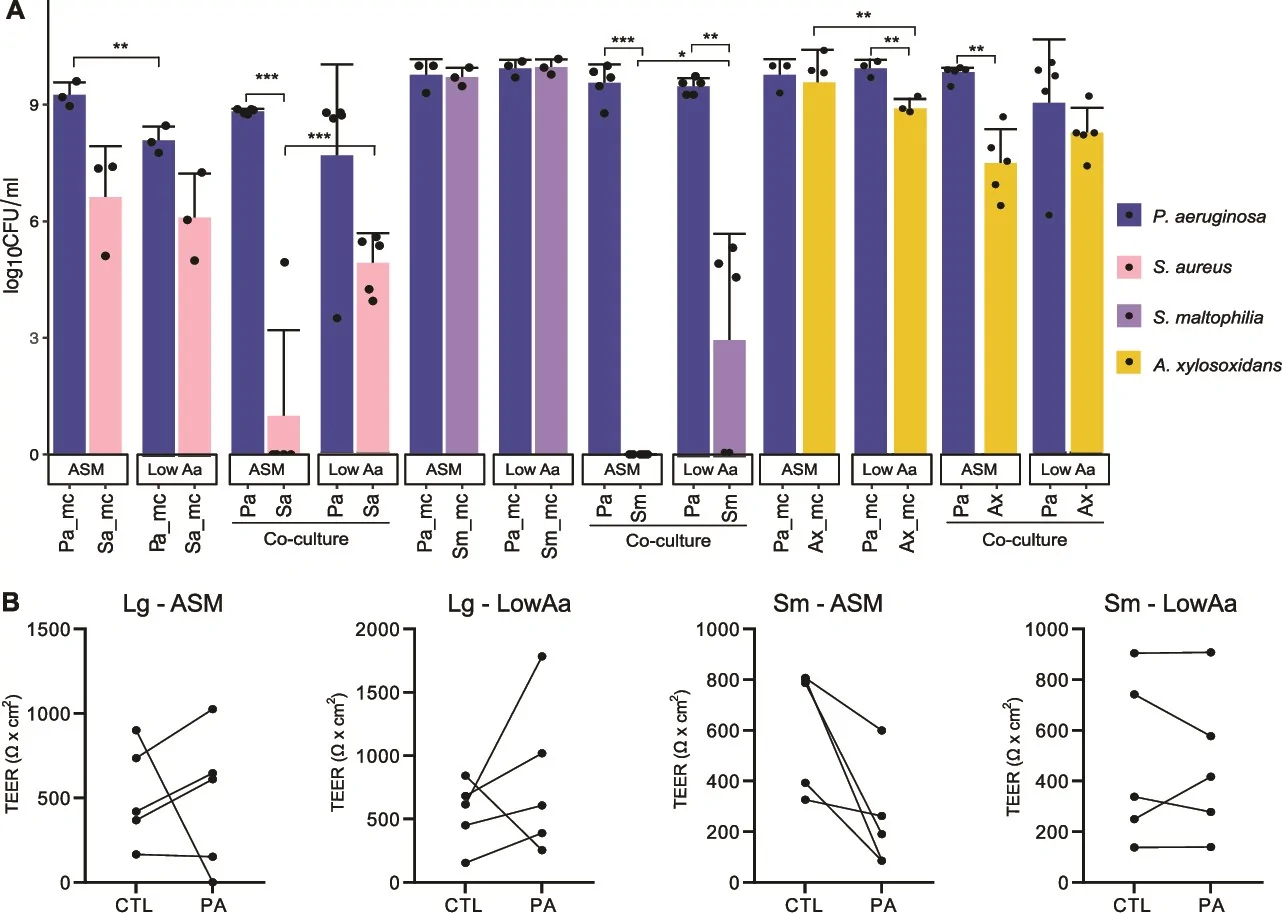
Growth under nutrient depletion impacts the virulence and microbial competition of *P. Aeruginosa*. (A) Growth in monoculture and co-culture experiments between *P. Aeruginosa* and other CF pathogens in ASM or ASM-LowAa conditions. Cell density was measured as log_10_(CFU/mL), and statistical significance was tested using the Student’s t-test. Barplots represent the mean, and error bars are the standard deviation. (B) TEER measurements of airway cells with culture supernatants from *P. Aeruginosa* grown under different conditions. Each dot represents the measurement taken from a single donor. The connecting line indicates paired data from the same donor with or without supernatant treatment from *P. Aeruginosa*. Supernatant treatment from *P. Aeruginosa* decreased TEER in small airways in ASM, but not in ASM-lowAa conditions. Where CTL means sterile media, and PA means *P. Aeruginosa* supernatant; statistical significance was tested using the paired t-test. Lg = large airway cells, Sm = small airway cells.

After finding that one of the strongest effects of the nutrient depletions to mimic ETI was altered *P. aeruginosa* virulence factor production, we tested whether growth from the organism under these nutrient-depleted conditions would alter its microbial competition and virulence. We focused on amino acid depletion because this has been directly demonstrated in vivo from metabolomics analysis of sputum [9], was repeatable in both the SBC and P1 sputum community experiment and amino acids are known to be precursors of many of the metabolites measured, particularly quinolones. Monoculture and co-culture experiments between *P. aeruginosa* and other CF pathogens, including *S. aureus*, *A. xylosoxidans*, and *A. maltophilia*, were performed in ASM or ASM-LowAa conditions, and growth was measured as colony-forming units per mL (CFU/mL). In monoculture, *P. aeruginosa* growth showed a slight detriment between ASM or ASM-LowAa (*P* < 0.01, Fig. 6A). *A. xylosoxidans* monoculture also showed reduced growth in the ASM-LowAa condition (*P* < 0.01). In co-culture, much of the microbial competition outcomes were altered between the ASM and ASM-LowAa conditions. *S. aureus* was almost completely killed when growing with *P. aeruginosa* in the ASM condition, but then its growth was rescued in co-culture in ASM-LowAa (*P* = 0.007). Similarly, *S. maltophilia* survived better grown with *P. aeruginosa* in the ASM-LowAa condition (*P* = 0.035). *A. xylosoxidans* showed increased growth in competition with *P. aeruginosa* in ASM-LowAa, but this did not reach statistical significance (*P* = 0.086, Fig. 6A). These experiments show that reduced amino acid concentrations in ASM, which are designed to mimic the effects of ETI on the sputum biochemistry, alter competition outcomes between *P. aeruginosa* and other pathogens.

To further explore the physiological consequences of nutrient depletions on *P. aeruginosa* virulence metabolites, we cultured the pathogen in monoculture under ASM and ASM-LowAa conditions, collected the cultures supernatants and used it to treat airway cells isolated from transbronchial and endobronchial biopsy samples for small and large airway epithelial cell cultures, respectively. These cells were collected from five different human volunteers, including separate isolation and growth of cells sourced from the lower and upper airways. After isolation, airway epithelial cells were expanded using established methods developed to conditionally reprogram them [37, 46]. Measuring TEER as an indicator of barrier integrity and cell survival showed that under ASM-LowAa conditions, the *P. aeruginosa* supernatants were less harmful to human airway cells. However, this phenomenon was only seen in cells isolated from the lower airways (Fig. 6B).

## Discussion

ETI treatment has resulted in significant improvement in the symptoms of CF, however, recent evidence indicates that microbial infection persists [10, 12, 16]. Paradoxically, improved airway clearance in pwCF has made it increasingly challenging to study the microbiology of lung infections due to the limited availability of the gold-standard clinical sputum sample [18]. This has created a need to develop new approaches to study these persistent infections and understand how the lung microbiome may change in response to this revolutionary therapeutic. Here we attempted to recapitulate the effects of ETI in an artificial microbial culture system by modulating the components of ASM and inoculating with CF sputum communities. Our manipulations of the microbiological media were based on early literature describing the molecular changes in airway mucus for those treated with ETI and a fundamental understanding of how improved CFTR function would be expected to alter mucus physiology and reduce pulmonary inflammation. Though this approach cannot perfectly mimic the complex changes in immunity, microbiology, mucus rheology, and biochemistry that have occurred in the lungs of pwCF on ETI, it does provide useful information about how a changing mucus environment will alter the behavior of CF pathogens, particularly *P. aeruginosa* [47–49].

As was expected, reducing concentrations of the major carbon sources provided to a complex microbial community altered its physiological function and generally reduced its growth. Unexpectedly, however, these experiments showed minimal changes in community structure, except when manipulating oxygen concentrations or reducing all carbon sources collectively to essentially starve the community. This supports the notion that the individual bacteria that comprise the lung microbiome may not change appreciably in those administered ETI, instead, their behavior and physiological functions could shift to a new state. This finding is supported in recent literature, where culture-independent and culture-dependent studies of the CF lung microbiome detected the same organisms prior to ETI treatment as afterward, and the community became more even in population distributions, but pathogen load was significantly reduced [3, 10–13, 16, 18, 39, 50].

The physiological changes observed here may reflect niche reforming and a subsequent shift in metabolism from community members. For example, even though nitrate concentrations were not altered in the ASM manipulations, nitrite reduction (as part of the microbial nitrite respiration pathway) was significantly different after community growth, even in samples where the community structure was not easily discernable between the ASM control and ndASM condition. This was observed in the amino acid-reduced ASM, where nitrite concentrations were high, but *P. aeruginosa* relative abundance changed minimally. Similarly, the SBC culture had a significant increase in pH to above 8 in conditions of lowDNA though the 16S rRNA genes microbiome profile showed only subtle changes. These chemical changes occurred in cultures of both the SBC and sputum communities of diverse composition, though not necessarily the same trends for each. These results indicate that changes in nitrogen metabolism and pH may be a unique consequence of ETI treatment on the lung microbiome, warranting future study of the inorganic chemistry within CF airway secretions. *P. aeruginosa* is a known denitrifying bacterium believed to use nitrate reduction as a primary mechanism of anaerobic respiration. The altered nitrite concentrations in the nutrient-depleted conditions with *P. aeruginosa* indicate that this metabolism may change with ETI.

The nutrient depletions also changed the microbial metagenome, with a particularly strong effect from a decreased DNA concentration. As inflammation in the airway improves and neutrophil recruitment is lower, it is expected that the DNA concentration of sputum will decrease as well, due to the limited production of neutrophil extracellular traps. The changes in pathway abundance in the sputum cultures mimicking this condition were somewhat unexpected and could have implications for the future of CF infections with lower neutrophil counts. Although the ndASM conditions had mostly subtle effects on community structure, incubation of cultures in oxic or anoxic conditions strongly altered the microbial community. This may be a major consequence of ETI treatment and possibly explain the increase in microbial evenness and diversity reported in other studies [8, 9, 12, 26]. As CFTR function improves and mucus is cleared out of the airways, it would be expected that oxygen penetration into the remaining mucus would be greater, with a potentially strong effect on microbial structure and function. Collectively, the multi-omics data and chemical measurements of our nutrient depletion experiments indicate that the community structure is mostly maintained, but there is a shift in community physiology.

One of the changes in our ndASM conditions with potential impacts on the health of pwCF, was a reduction in metabolites associated with virulence and microbe-microbe competition from *P. aeruginosa*. This was especially repeatable under ASM-LowAa conditions, a known effect of ETI on sputum biochemistry [9], where quinolones, rhamnolipids, and phenazines were reduced. It is likely that the reduction in quinolones, and indirectly, rhamnolipids and phenazines due to their regulation by quorum sensing, is because certain amino acids are the precursors of their synthesis. Quorum sensing allows *P. aeruginosa* to adapt strategically to its own cell density, enhancing its survival and infection capabilities [51, 52]. Rhamnolipids and phenazines are secondary metabolites regulated by quorum sensing systems. Thus, quorum sensing functions as a population density-dependent regulatory network, ensuring the production of these molecules only when bacterial populations reach a sufficient density [53, 54]. This coordination optimizes their effectiveness in ecological and pathogenic processes, such as biofilm formation, interspecies competition, and virulence [42, 43]*.* It has long been known that quinolone production by *P. aeruginosa* is stimulated by the presence of aromatic amino acids in the growth medium and occurs via anthranilate as a biochemical precursor [40, 41]. A similar phenomenon is likely occurring here, where reduction in the amino acid component of ASM, including those with aromatic residues, results in reduced production of quinolones through the anthranilate pathway, which subsequently reduces rhamnolipid and phenazines in the culture medium. It is important to note, that the SBC total bacterial load decreased by approximately 1.5 log CFU/mL under the ASM-LowAa condition, which could also contribute to lower amounts of these molecules. However, other metabolites from the bacterium were not significantly different (eg. pyochelin), this did not occur with other ndASM conditions, and a pure culture of *P. aeruginosa* under the same conditions had only a slight growth detriment.

Because of the effect of reduced quinolone and rhamnolipid production from the nutrient depletions, we investigated the consequences of this on microbe-microbe competition and *P. aeruginosa* virulence. It was found that under the ASM-LowAa conditions, *P. aeruginosa* exhibited less killing of competing pathogens, particularly *S. aureus* and *S. maltophilia*. The former CF pathogen has been shown to persist in the airways of pwCF on ETI from clinical culture results, more so than that of *P. aeruginosa* [49]. Our experiments provide a possible mechanism for *S. aureus* persistence in those on ETI due to improved competition outcomes with *P. aeruginosa*, though this is only speculative and will require further study. The mechanism of this in vitro rescue in co-culture with *P. aeruginosa* may be due to the reduction in quinolones under ASM-LowAa conditions, which have been shown to kill *S. aureus* and disrupt its aerobic respiration in co-culture. Regardless of the mechanism of action, these results are indirect evidence that the microbial ecology and competition within the airways of pwCF has changed on ETI, which will require more direct study in the airways to understand the implications for disease pathophysiology.

The *P. aeruginosa* virulence metabolites not only affect competing pathogens but can also harm human cells [55–58]. We therefore tested whether *P. aeruginosa* grown in ASM-LowAa conditions could alter its impact on human lung epithelial cells isolated and cultured from explant human lung tissues. When exposing these epithelial cells to culture supernatants from *P. aeruginosa* in the ASM and ASM-LowAa conditions we found that the latter reduced the epithelial barrier integrity, an indicator of reduced cell viability and increased cell damage. This only occurred in human cells isolated from the lower airways, meaning that the negative impacts of the bacterium in pwCF may be most significant deeper in the lung bronchial tree. These experiments provide indirect evidence that the consequences of a non-antibiotic therapy designed to improve CFTR function on mucus biochemistry can alter the virulence of *P. aeruginosa* in CF airways. This will require a more direct study to determine if this is occurring in vivo, but if true, it may represent an unexpected benefit to pwCF taking ETI.

Collectively, this study foreshadows the changes in CF lung microbiome structure and function in the ETI era of CF disease, which has become much more challenging to study due to the reduction in sputum production. Though these findings provide only a picture of the changing CF lung infections, they highlight focus areas of CF pathophysiology that require more direct study including changes in microbiome physiology, inorganic chemistry, and virulence factor production in the novel ETI environment.

## Supplementary Material

Supp_mat_correctionsv4_CTG_wraf125

## Data Availability

The data generated and analyzed during this study are publicly available for Microbiome access through the Qiita studies CF_SBC.4.23 (ID:14987, https://qiita.ucsd.edu/study/description/14987) and for pwCF samples P1-P7 (ID: 14985, https://qiita.ucsd.edu/study/description/14985). All raw sequencing data generated in this study have been deposited in the NCBI Sequence Read Archive under BioProject accession numbers PRJNA1264509 and PRJNA1263877. The metabolomic job is available at https://gnps.ucsd.edu/ProteoSAFe/status.jsp?task=52b158ef6f3a48fb800a9bf311035c38.
