# Supplementary material for: Microenvironmental effects of a non-antibiotic therapy for a chronic Polymicrobial infection Alter microbial physiology, competition, and virulence
Source: ISME J. 2025 Jun 14;20(1):wraf125. doi: 10.1093/ismejo/wraf125 (PMC13248941; doi:10.1093/ismejo/wraf125)
Supplement: Supp_mat_correctionsv4_CTG_wraf125 [file supp_mat_correctionsv4_ctg_wraf125.pdf]

Supplementary information for

Microenvironmental Effects of a Non-antibiotic Therapy for a Chronic Polymicrobial Infection Alter Microbial Physiology, Competition, and Virulence

Cely T. González[1], Christian Martin[1], Maddey Crane[1], Karen Gutierrez[1], Jacob Thomas[1], Lacy Remisoski[1], Maxwell Okros[1], Yousi Fu[1], Douglas V. Guzior[1,2], Dustin Finkhouse[1], Christopher Bridges[1], Jenna Mielke[3], Gabriel Querido[3], Lienwil Padillo[3], Reda Girgis[4], Marc McClelland[4], Douglas Conrad[3], Xiaopeng Li[5] and Robert A. Quinn[1*]

[1] Department of Biochemistry and Molecular Biology, Michigan State University, East Lansing, MI, USA.

[2] Department of Microbiology, Genetics and Immunology, Michigan State University, East Lansing, MI, USA.

[3] Department of Medicine, University of California San Diego, La Jolla, CA, USA.

[4] Corewell Health, Grand Rapids, MI, USA.

[5] Department of Pediatrics and Human Development, Michigan State University, East Lansing, MI, USA

This PDF file includes:

Supplementary Tables 1 to 10

Supplementary Figures 1 to 11

Supplemental material

Table S1. Artificial Sputum Media (ASM) formula preparation and proportions used in this study to carbon sources like nutrient-depletion (ndASM) Amino acid (Aa), DNA, and Mucin.

Item	mL (ASM)[20,22#]	ml (ndASM) LowAa	ml (ndASM) LowDNA	ml (ndASM) LowMucin
5% mucin stock (50 mg/ml)	8ml	8ml	8ml	*0.8ml
KCl stock (44 mg/ml)	1ml	1ml	1ml	1ml
NaCl stock (100 mg/ml)	1ml	1ml	1ml	1ml
Egg yolk emulsion	0.1ml	0.1ml	0.1ml	0.1ml
#Salmon sperm DNA (10 mg/ml)	2.8ml	2.8ml	*0.28ml	2.8ml
MEM non-essential amino acid mix (100X)	2.89ml	*0.289ml	2.89ml	2.89ml
MEM amino acid mix (50X)	2.89ml	*0.289ml	2.89ml	2.89ml
Sterile water	0.001ml	0.001ml	0.001ml	0.001ml
Ferritin (1mg/mL stock)	0.06ml	0.06ml	0.06ml	0.06ml
Phenol red stock (4 mg/ml)	0.2ml	0.2ml	0.2ml	0.2ml
Phosphate-Buffered Saline	0ml	5.202ml	2.52ml	7.2ml
Total volume	18.94ml	18.94ml	18.94ml	18.94ml

*Denotes the carbon source diluted one at a time for each nutrient depletion experiment in 5 replicates per each. To reach the total volume, phosphate-buffered saline (PBS) was used to compensate.

"Tucker et al. (2021) demonstrated that in the βENaC-Tg animal model, neutrophils are the predominant inflammatory cells, and their breakdown releases high DNA concentrations along with amino acids nutrient sources that bacteria can readily utilize for growth in CF."

Table S2. General features of the bacterial isolates in the Synthetic Bacterial Community (SBC) utilized for nutrient depletion experiments.

Bacteria	O_2 Growth Conditions	Aerotolerance	Respiration	Fermenter	Selective media
**Pseudomonas aeruginosa*	Aerobe	Facultative anaerobe	+	-	Pseudomonas Isolate Agar (PIA)
**Achromobacter xylosoxidans*	Aerobe	Facultative anaerobe	+	-	Tryptic Soy Agar (TSA)/ McConkey Agar (MCA)
**Stenotrophomonas maltophilia*	Aerobe	Facultative anaerobe	+	-	PIA/Brain Heart Infusion agar (BHI)
**Staphylococcus aureus*	Anaerobe	Facultative anaerobe	+	+	TSA/Mannitol Salt Agar (MSA)
**Rothia mucilaginosa*	Aerobe	Facultative anaerobe	+	+	TSA supplemented/Brucella agar (BRU broth)
Prevotella melaninogenica	Anaerobe	Strict anaerobe	-	+	Chopped Meat with Carbohydrates Media (CMC)/ BRU broth
**Veilonella atypica*	Anaerobe	Strict anaerobe	-	+	Reinforced Clostridium Medium (RCM)
**Streptococcus gordonii*	Aerobe	Facultative anaerobe	+	+	TSA supplemented/ BHI
Granulicatella elegans	Anaerobe	Facultative anaerobe	+	+	TSA supplemented/ RCM

*Denotes the pure isolates from pwCF.

Tryptic Soy Agar supplemented with vitamin B6 and/or defibrinated sheep blood 5%

Table S3. Clinical characteristics of fresh sputum samples from pwCF in this study.

Sputum Sample	Age	Trikafta (Yes/No)	Gender	*FEV1 PP	*FEV1	Clinic Culture Results	Patients Status
P1	49	N	Female	40%	1.23	*Mycobacteriodes abscessus, Aspergillus fumigatus*, ESBL *Escherichia coli, Pseudomonas aeruginosa, Scedosporium apiospermum*	Stable
P2	32	Y	Female	75%	2.50	Yeast, not Cryptococcus species; *Moraxella catarrhalis; Haemophilus influenzae*	Stable
P3	55	Y	Female	64%	1.74	Yeast, not Cryptococcus species; *Moraxella catarrhalis; Haemophilus influenzae*	Exacerbation
P4	35	Y	Male	77%	2.98	Moderate Normal Respiratory Flora; *Pseudomonas aeruginosa,* one colony; Yeast, not Cryptococcus species	Stable
P5	76	N	Male	31% (6/22/2022)	1.06 (6/22/2022)	Mucoid *Pseudomonas aeruginosa*; Yeast, not Cryptococcus species	Exacerbation
P6	33	N	Male	80%	3.25	Yeast, not Cryptococcus species(4/18/2022); *Stenotrophomonas maltophilia*; *Scedosporium apiospermum*; Yeast, not Cryptococcus species (8/26/2022)	Exacerbation
P7	76	N	Male	34% (7/6/2022)	1.18 (7/6/2022)	*Mycobacterium lentiflavum*; *Achromobacter xylosoxidans*; Mucoid *Pseudomonas aeruginosa*; Yeast, not Cryptococcus species	Exacerbation

*FEV1 PP: Forced Expiratory Volume Percent Predicted.
*FEV1: Forced Expiratory Volume in One Second.

Table S4.
Confusion matrix from Random Forest classification of sputum ASM and nutrient-depleted ASM cultures.
This table compares the predicted versus actual classifications under nutrient-depleted conditions, showing the number of correctly and incorrectly classified instances for each culture type.
Out-of-bag error rate 64.29%

	LowAa	ASM	LowDNA	LowMu	class.error
LowAa	1	4	1	1	0.8571429
ASM	4	1	1	1	0.8571429
LowDNA	0	2	5	0	0.2857143
LowMu	3	0	1	3	0.5714286

Table S5. Variable importance plot from metagenome RF of aerobic sputum communities.

Humann Pathway	Mean Decrease Accuracy (%)
PPGPPMET.PWY..ppGpp.metabolism	13.399162
PWY.6396..superpathway.of.2.3.butanediol.biosynthesis	11.1894538
PWY.7198..pyrimidine.deoxyribonucleotides.de.novo.biosynthesis.IV	10.7780582
PWY.7328..superpathway.of.UDP.glucose.derived.O.antigen.building.blocks.biosynthesis	10.3954873
PWY66.399..gluconeogenesis.III	10.356015
PWY.7383..anaerobic.energy.metabolism..invertebrates..cytosol.	10.073383
PWY.7210..pyrimidine.deoxyribonucleotides.biosynthesis.from.CTP	10.0578156
ILEUSYN.PWY..L.isoleucine.biosynthesis.I..from.threonine.	8.98530017
PWY.5103..L.isoleucine.biosynthesis.III	8.30519418
PWY.7184..pyrimidine.deoxyribonucleotides.de.novo.biosynthesis.I	6.7817613
PWY.6797..6.hydroxymethyl.dihydropterin.diphosphate.biosynthesis.II..Methanocaldococcus.	6.62801313
PWY.7663..gondoate.biosynthesis..anaerobic.	6.48195234
PWY.5973..cis.vaccenate.biosynthesis	6.11747719
PHOSLIPSYN.PWY..superpathway.of.phospholipid.biosynthesis.I..bacteria.	5.95641513
NONMEVIPP.PWY..methylerythritol.phosphate.pathway.I	5.73767826
BRANCHED.CHAIN.AA.SYN.PWY..superpathway.of.branched.chain.amino.acid.biosynthesis	5.73192992
PWY.5837..2.carboxy.1.4.naphthoquinol.biosynthesis	5.39246824
PWY.5121..superpathway.of.geranylgeranyl.diphosphate.biosynthesis.II..via.MEP.	5.01689951
PWY.6471..peptidoglycan.biosynthesis.IV..Enterococcus.faecium.	4.97833961
PWY.7111..pyruvate.fermentation.to.isobutanol..engineered.	4.88743978
CALVIN.PWY..Calvin.Benson.Bassham.cycle	4.71493096
UDPNACETYLGALSYN.PWY..UDP.N.acetyl.D.glucosamine.biosynthesis.II	4.47159157
PWY.6609..adenine.and.adenosine.salvage.III	4.41025635
PWY.6147..6.hydroxymethyl.dihydropterin.diphosphate.biosynthesis.I	4.1385394
SER.GLYSYN.PWY..superpathway.of.L.serine.and.glycine.biosynthesis.I	4.00855956
PWY.621..sucrose.degradation.III..sucrose.invertase.	3.79735835
VALSYN.PWY..L.valine.biosynthesis	3.76788915
PWY.5659..GDP.mannose.biosynthesis	3.67954915
PWY0.1298..superpathway.of.pyrimidine.deoxyribonucleosides.degradation	2.83081664
PWY.6823..molybdopterin.biosynthesis	2.82898444

Table S6. Confusion matrix from RF classification of sputum ASM and ndASM cultures based on nutrient-depleted condition.

Out of bag error = 0%

Actual/Predicted	Aerobe	Anaerobe	class.error
Aerobe	18	0	0.00
Anaerobe	0	20	0.00

Table S7. Variable importance plot from the metabolome RF of aerobic sputum communities.

**Metabolite**	**Mean Decrease Accuracy (%)**
X5576_555.392568718002_6.512952_N.A	8.00034325
X4885_216.138293127714_5.027985_N.A	7.45637102
X5172_159.066160718012_5.824144_N.A	7.40457853
X4884_260.163851348369_5.025668_N.A	7.40194916
X4939_228.138237023288_5.1986284_N.A	7.35268025
X5143_288.194976311779_5.7600427_2..hydroxy.2.nonyl.4.quinolone..2..OH.NQ.	7.23636667
X5044_242.153103153543_5.456225_HHQ.db	7.17668035
X5157_487.331822205007_5.7992353_N.A	6.98964152
X5163_244.168753950111_5.8063245_2.heptylquinolin.4.1H..one	6.97111051
X5023_230.159785709888_5.3913035_N.A	6.93960454
X2596_938.454310608804_3.4956415_N.A	6.84175479
X5049_282.147534873235_5.4864225_N.A	6.81670822
X1533_540.772223358749_3.0820234_N.A	6.80913032
X5907_324.232119578538_7.0678544_N.A	6.69869795
X5151_268.17249422078_5.7907763_N.A	6.6766852
X1704_641.805276048539_3.1416423_N.A	6.64983352
X1607_399.708721870298_3.1118217_N.A	6.6489398
X5302_270.184364123737_6.1030693_2..2.nonen.1.yl..4.Quinolinol	6.64236809
X1558_490.251564365558_3.091904_N.A	6.55280173
X5145_254.153259362972_5.764233_N.A	6.52423737
X6184_668.420562270733_7.5267262_N.A	6.51343911
X6474_354.279538186746_7.9537115_N.A	6.49905899
X6577_696.454065264139_8.102374_N.A	6.49356218
X3784_162.055083168036_4.089362_4.hydroxy.2.quinolone.CollisionEnergy.205060	6.44888181
X5374_299.304831025938_6.1988297_N.A	6.34953841
X6392_543.291606443786_7.8321605_N.A	6.33553016
X6568_701.404861153438_8.092519_N.A	6.29269877
X5534_184.073788184744_6.467426_N.A	6.28523321
X1644_721.862641256161_3.121426_N.A	6.26512233
X5275_297.289456030152_6.0272326_N.A	6.24960513

Table S8. Confusion matrix from RF classification of sputum ASM and ndASM cultures in aerobic conditions based on nutrient-depleted condition.
Out of bag error = 27.7%

Actual/Predited	ASM-LowAa	ASM	ASM-LowDNA	ASM-LowMu	class.error
ASM-LowAa	4	1	0	0	0.2
ASM	0	16	1	1	0.1111111
ASM-LowDNA	0	1	4	0	0.2
ASM-LowMu	0	4	1	0	1

Table S9. Variable importance plot from the metabolome RF of aerobic sputum communities.

Metabolite	Mean Decrease Accuracy (%)
X6239_664.463191703458_7.5772047_N.A	6.674511179
X2189_895.397101666174_3.340684_N.A	6.202441501
X427_331.165297341346_1.3663356_Spectral.Match.to.DL.Phenylalanine.from.NIST14	5.81929537
X3348_643.32881284974_3.840369_N.A	5.58892167
X5049_282.147534873235_5.4864225_N.A	5.551223537
X1462_561.782748388275_3.0418992_N.A	5.450391723
X4763_308.221006718056_4.8184953_N.A	5.421716905
X3260_805.432217447034_3.7984169_N.A	5.375631752
X850_146.117594553416_2.3136907_N.A	5.345903285
X1241_304.177067406267_2.944952_N.A	5.337199372
X1045_409.186705261598_2.8245342_N.A	5.231010794
X4655_269.135734377639_4.6971292_N.A	5.05511355
X446_207.112869191412_1.3750887_N.A	4.990653392
X1031_132.080959761853_2.8161426_NORLEUCINE	4.930057241
X3096_377.145154765087_3.709544_....Riboflavin...40.0.eV	4.920027977
X3583_631.328005867718_3.9725673_N.A	4.825573302
X3293_966.437616656743_3.8125134_N.A	4.725820847
X1374_690.313940320355_3.0087361_N.A	4.69616294
X886_409.187276591621_2.4060457_N.A	4.676074301
X4063_643.305134510893_4.2756286_N.A	4.664317988
X4895_341.060797157633_5.046838_N.A	4.641067024
X6292_522.353571609555_7.6536927_Spectral.Match.to.1..9Z.Octadecenoyl..sn.glycero.3.phosphocholine.from.NIST14	4.569383255
X6762_697.491138795614_8.379934_N.A	4.56309397
X5979_468.366286414561_7.1912537_N.A	4.477948738
X6178_409.234899463061_7.5220633_N.A	4.477766578
X3795_354.079754741254_4.097682_N.A	4.476027316
X5764_583.424940981228_6.812212_N.A	4.345901538
X1023_188.070598688249_2.8105574_Spectral.Match.to.Abrine.from.NIST14	4.230989752
X1029_118.06517919756_2.8155534_N.A	4.21209786
X7116_689.491830897796_8.914202_N.A	4.188552285

Table S10. pH measurement for SBC using RGB color.

Treatment	SBC RBG Color Sum	SBC pH
ASM	117883	6.3780727
ASM	125412	6.1288628
ASM	122448	6.2269712
ASM	97673	7.0470237
ASM	93873	7.1728037
Control	80327	7.6211763
Control	66928	8.0646832
Control	77032	7.7302408
Control	65609	8.1083421
Control	76923	7.7338487
Control	101789	6.9107841
LowAA	119111	6.3374259
LowAA	121139	6.2702991
LowAA	113394	6.5266586
LowAA	98527	7.0187563
LowAA	90359	7.2891171
LowDNA	77979	7.6988951
LowDNA	64553	8.1432957
LowDNA	56625	8.4057125
LowDNA	73251	7.8553919
LowDNA	54372	8.4802868
LowMu	101121	6.9328949
LowMu	110400	6.62576
LowMu	86758	7.4083102
LowMu	88513	7.3502197
LowMu	87777	7.3745813

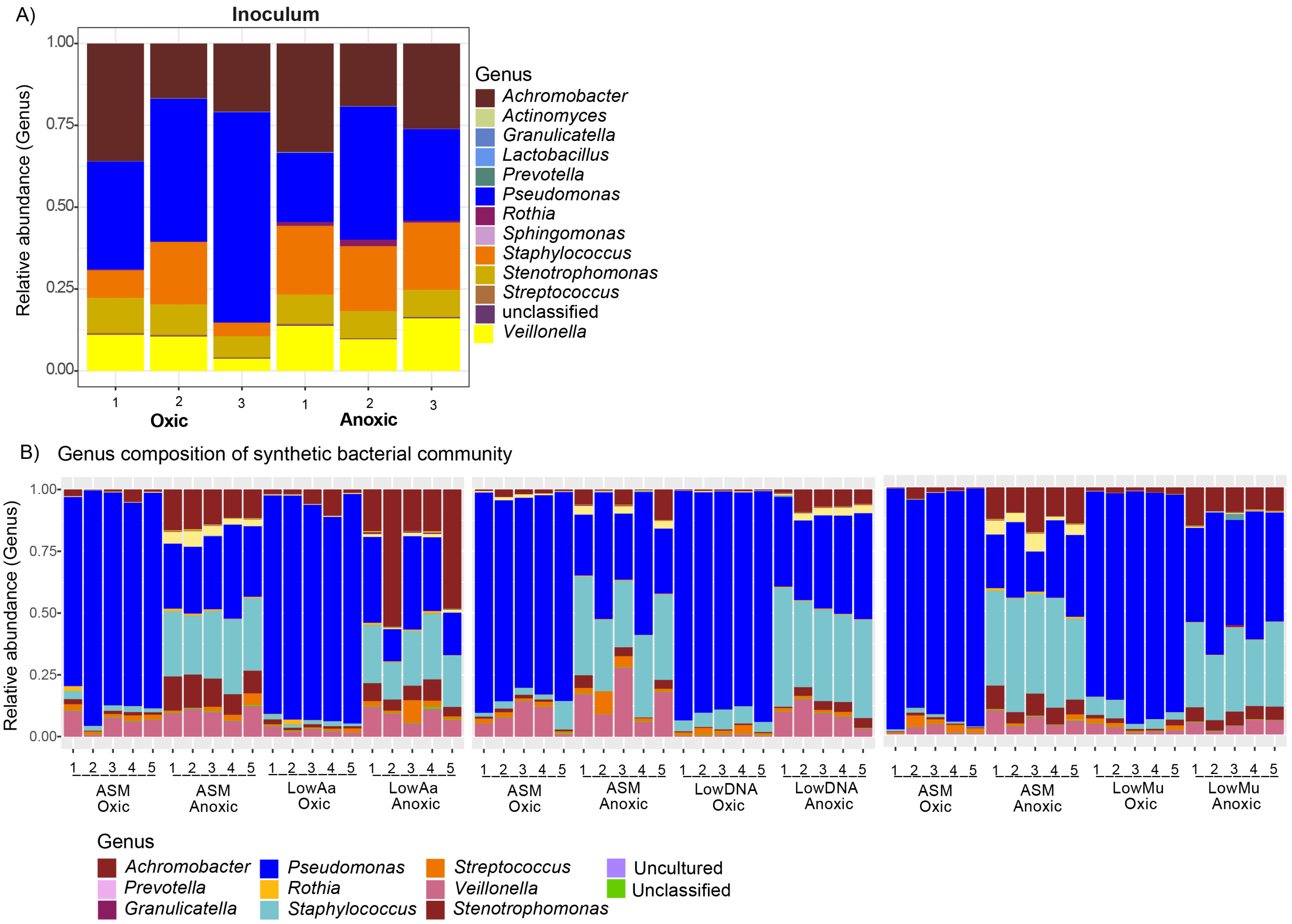

Figure S1. 16S rRNA gene sequencing results of SBC inoculum assembly after 24 hours and in the nutrient depletion experiments. a) Microbiome profile of the the nine-member community after plate inoculation and 24 hour growth in ASM in aerobic and anaerobic conditions. This starter community used as an inoculum for the nutrient depletion experiments. b) Microbiome profiles of each individual replicate (n = 5) for each nutrient depletion condition with the SBC.

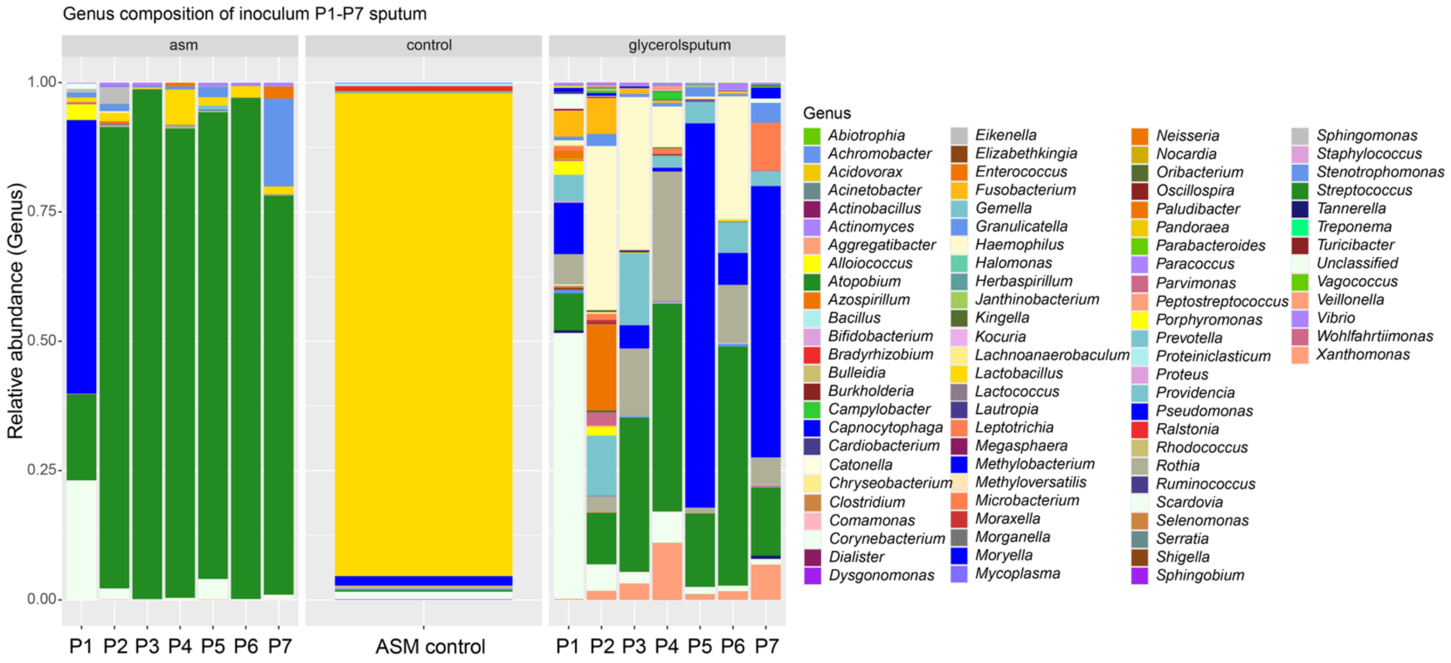

Figure S2. Barplot of genus composition of patient sputum samples prior to and after culture in ASM. .

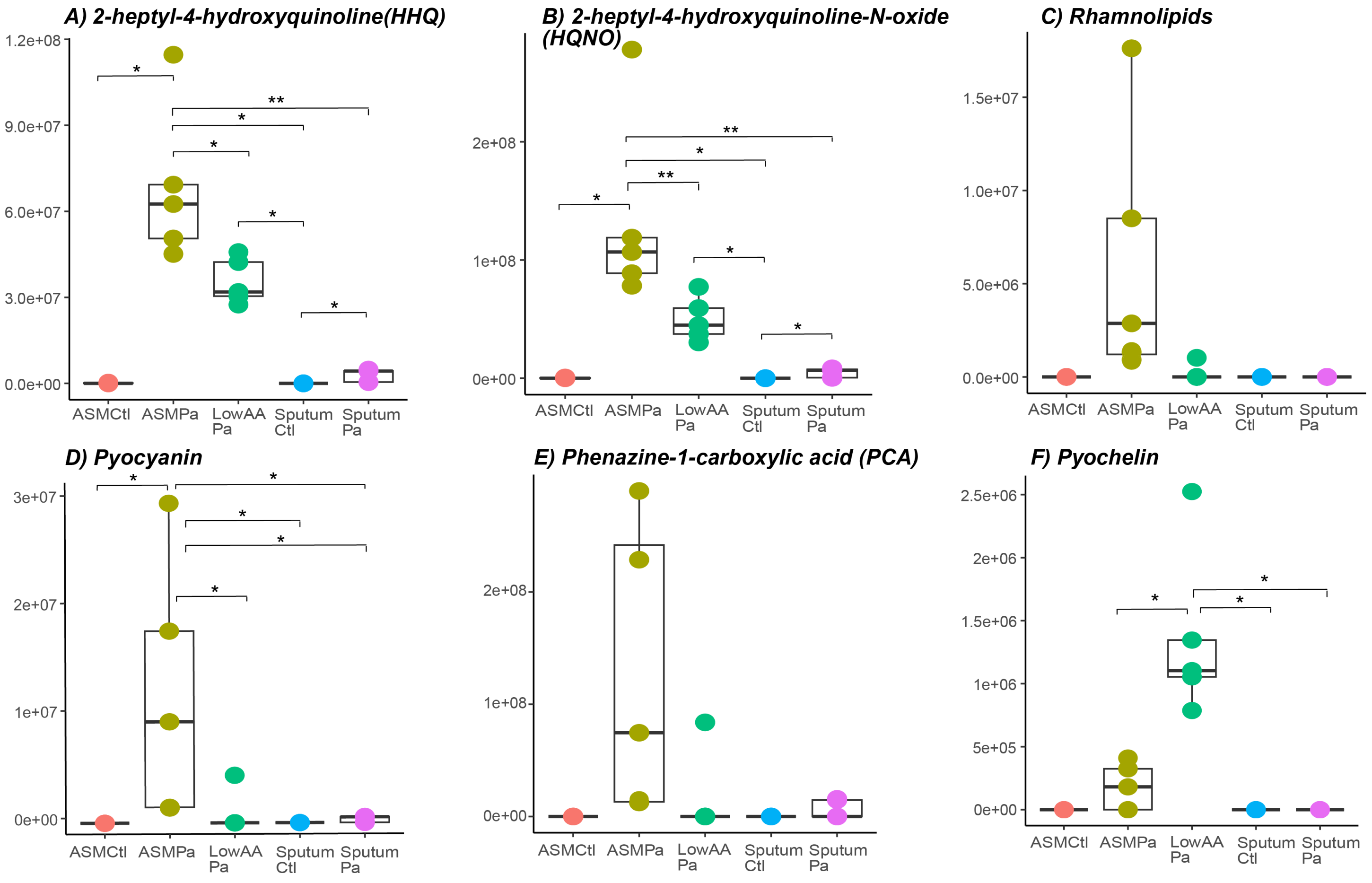

Figure S3. Media nature effects on *P. aeruginosa* virulence metabolites.

Relative levels of (a) 2-heptyl-4-hydroxyquinoline (HHQ), (b) 2-heptyl-4-hydroxyquinoline-N-oxide (HQNO), (c) rhamnolipids, (d) pyocyanin, (e) phenazine-1-carboxylic acid (PCA), and (f) pyochelin in *P. aeruginosa* monoculture

under ASM, fresh sputum and ASM-LowAa conditions are driven by nutrient depletion. Statistical significance between conditions was determined using Kruskal–Wallis with Dunn's multiple comparisons, where $p < 0.05$ is * and $p < 0.01$ is **. The ASM-LowAa and ASM can alter the production of these virulence factors.

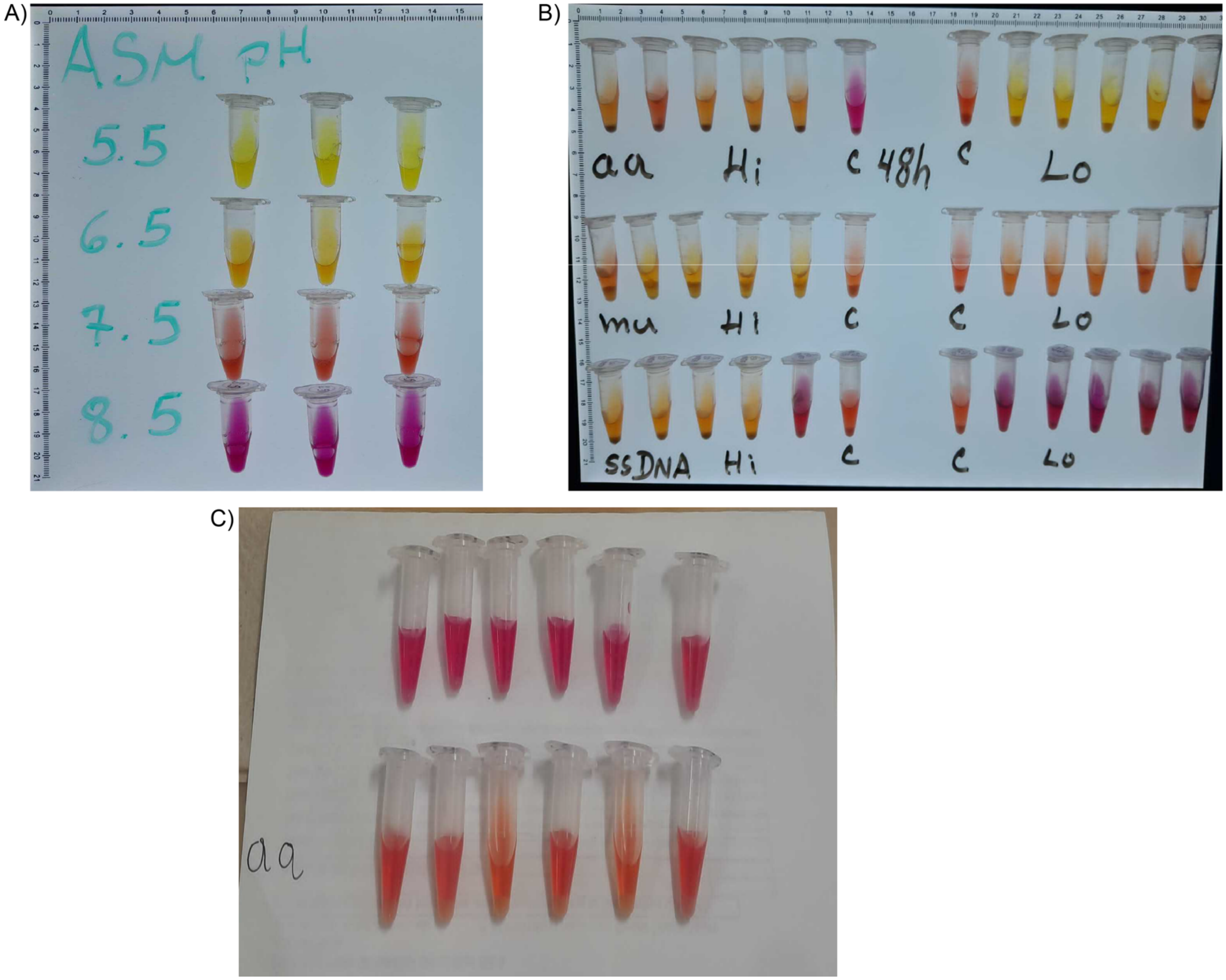

Figure S4. Nutrient depletion pH shift using phenol red dye in oxygenic conditions experiment. a) Images of the tube in different pH to build a standard curve. b) SBC tubes in ASM media and ndASM (LowAa, LowDNA, LowMu) in aerobic conditions were captured with a camera after 48h using a lightbox.

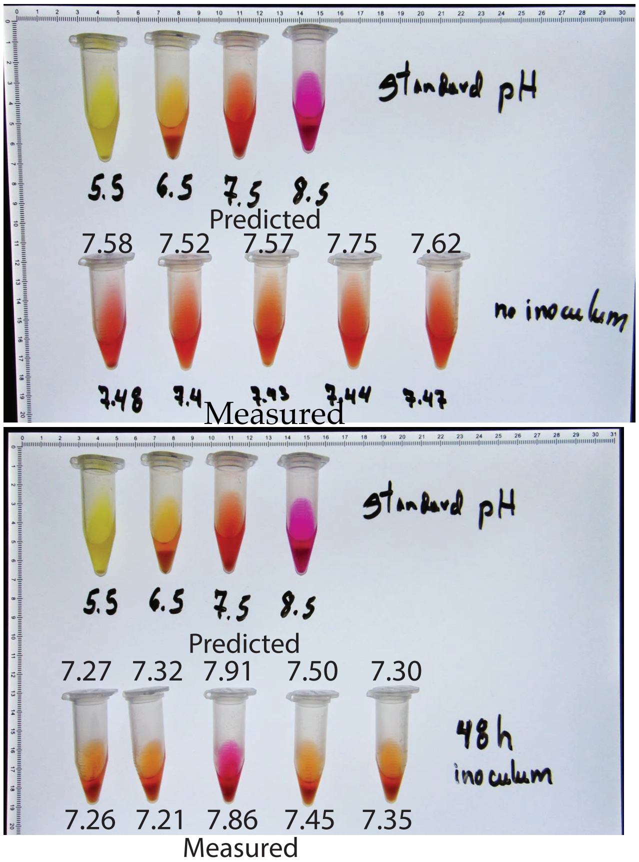

Figure S5. Image-based pH prediction, prediction accuracy. a) pH standards from 5.5 to 8.5 range, compared with the image of steril ASM media before inoculation. Image predicted pH and measured pH are shown. b) inoculated cultures after 48 hours compared with the standards. The error range was +/-0.17 pH units for the former and 0.07 pH units for the latter.

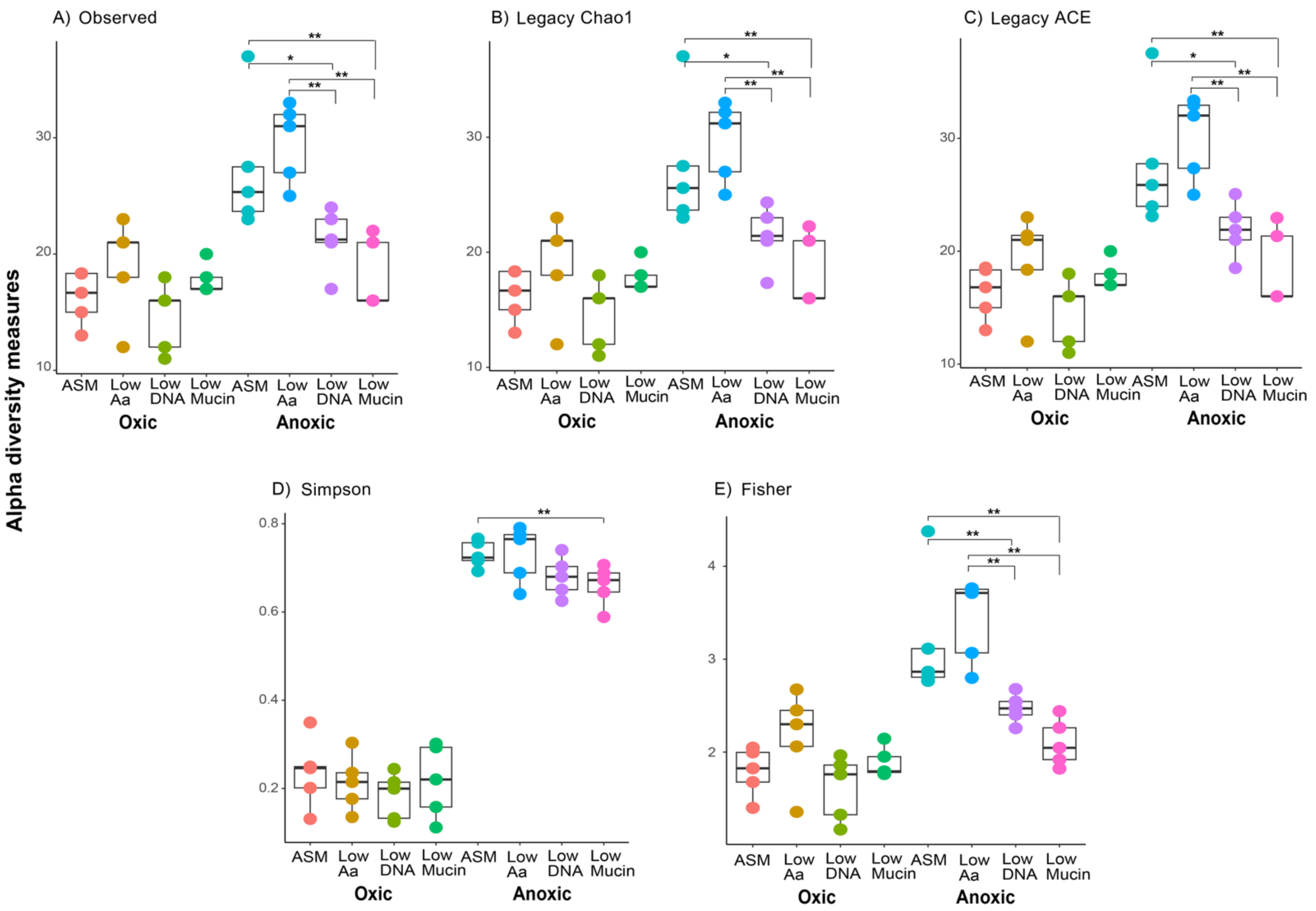

Figure S6. Alpha Diversity Measures for SBC under aerobic and anaerobic conditions with different growth media compositions: artificial sputum media (ASM), low amino acids (Low Aa), low DNA (Low DNA), and low mucin (Low Mucin). a) Alpha diversity applies to the observed richness; anaerobic conditions generally show higher richness than aerobic conditions. b) An estimate of species richness based on rare species, demonstrating significant increases under anaerobic conditions, particularly in low-amino-acid media. c) The abundance-based coverage estimator highlights diversity differences, with anaerobic conditions showing significantly higher values. d) The Simpson index quantifies diversity by incorporating evenness, indicating lower evenness under anaerobic conditions. e) Fisher's alpha diversity metric shows a richness difference under anaerobic conditions among nutrient depletion. Boxplots display diversity indices across experimental replicates, highlighting significant differences between conditions as denoted by asterisks (*$p < 0.05$; **$p < 0.01$).

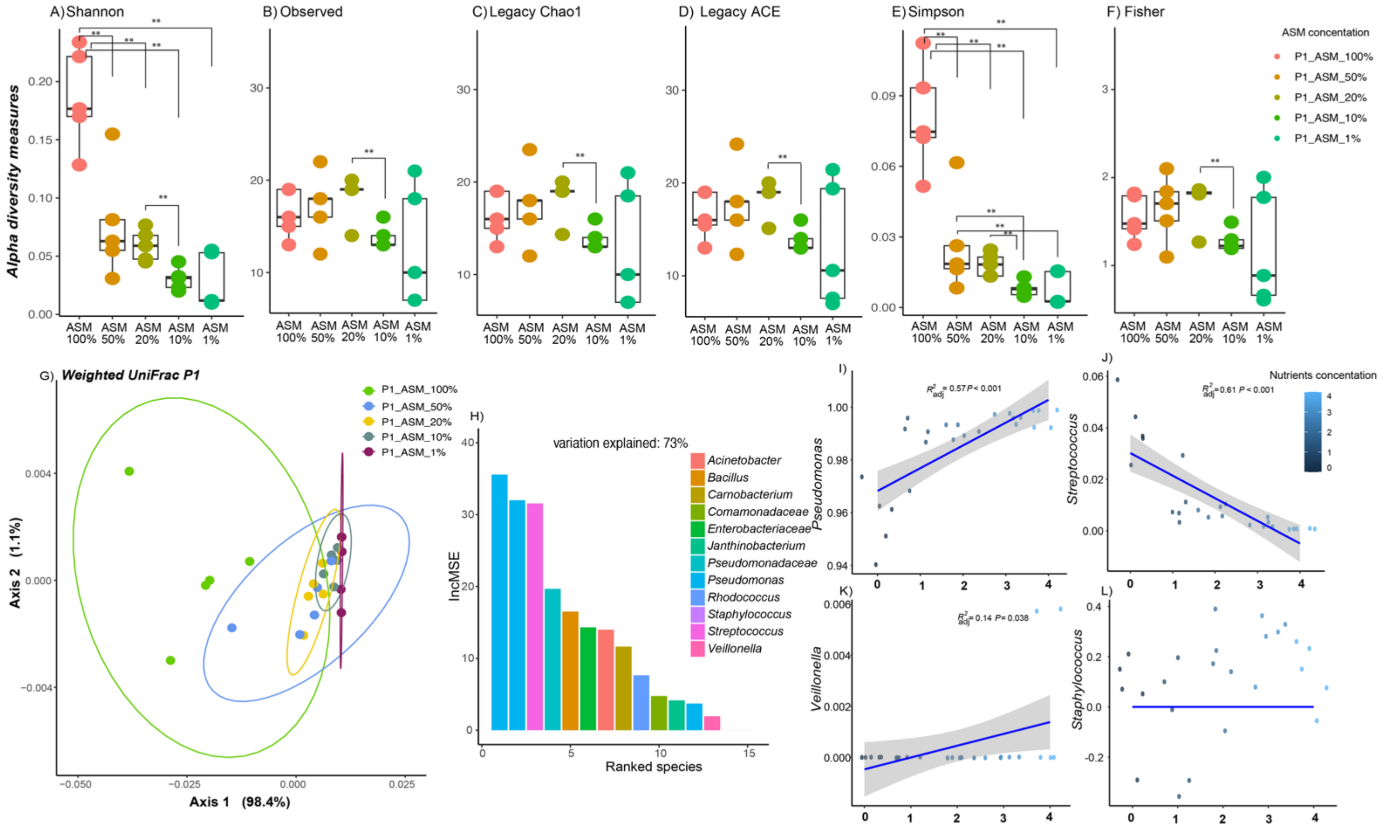

Figure S7. Alpha diversity, beta diversity, and microbial taxonomic responses of fresh sputum P1 communities across the experiment, where all nutrients were depleted. (a–f) Boxplots showing alpha diversity metrics, including Shannon index and observed ASVs (a-d), Simpson (e), and Fisher (f) indices across artificial sputum media (ASM) dilutions (ASM100%, ASM50%, ASM20%, ASM10%, ASM1%). g) Weighted UniFrac principal coordinate analysis (PCoA) illustrating beta diversity of the P1 community across ASM dilutions. Clustering patterns demonstrate the impact of nutrient availability on community structure. PERMANOVA tests indicated significant differences in the beta diversity of SBC nutrient depletion across the ASM full and the other dilutions (Adonis: Weighted Unifrac, R^2=0.80, p<0.05, F=21.06). (h) Feature importance plot from Random Forest regression analysis showing the explanatory power of key bacterial species for the observed variations in community composition. Bars represent the % variance explained by each species, with the model accounting for 73% of the total variance. (i–l) Linear regression plots depict relationships between ASM serial dilutions and the relative abundances of specific bacterial taxa: *Pseudomonas*, *Streptococcus, Veillonella,* and *Staphylococcus*. Regression equations, adjusted R^2 values. Shaded regions represent 95% confidence intervals. Significant differences were determined using pairwise statistical comparisons (*p < 0.05, **p < 0.01, ***p < 0.001).

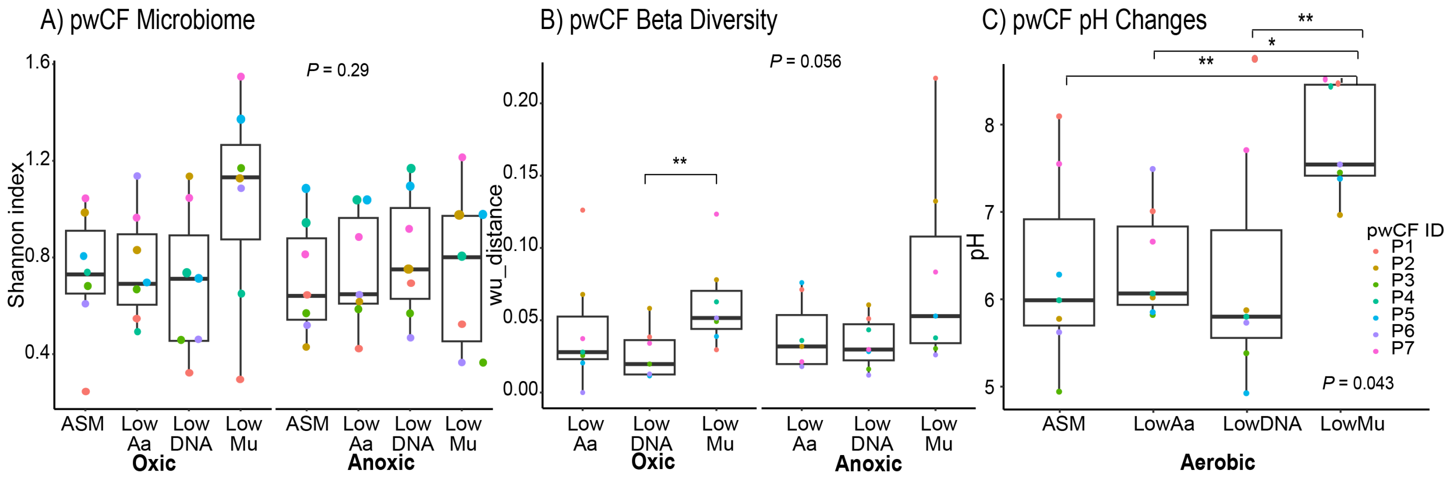

Figure S8. pwCF P1-P7 fresh sputum analysis (n = 7). a) Alpha-diversity across the seven sputum communities (patients) in the nutrient depletion experiment, b) beta-diversity compared to ASM control and the c) pH changes.

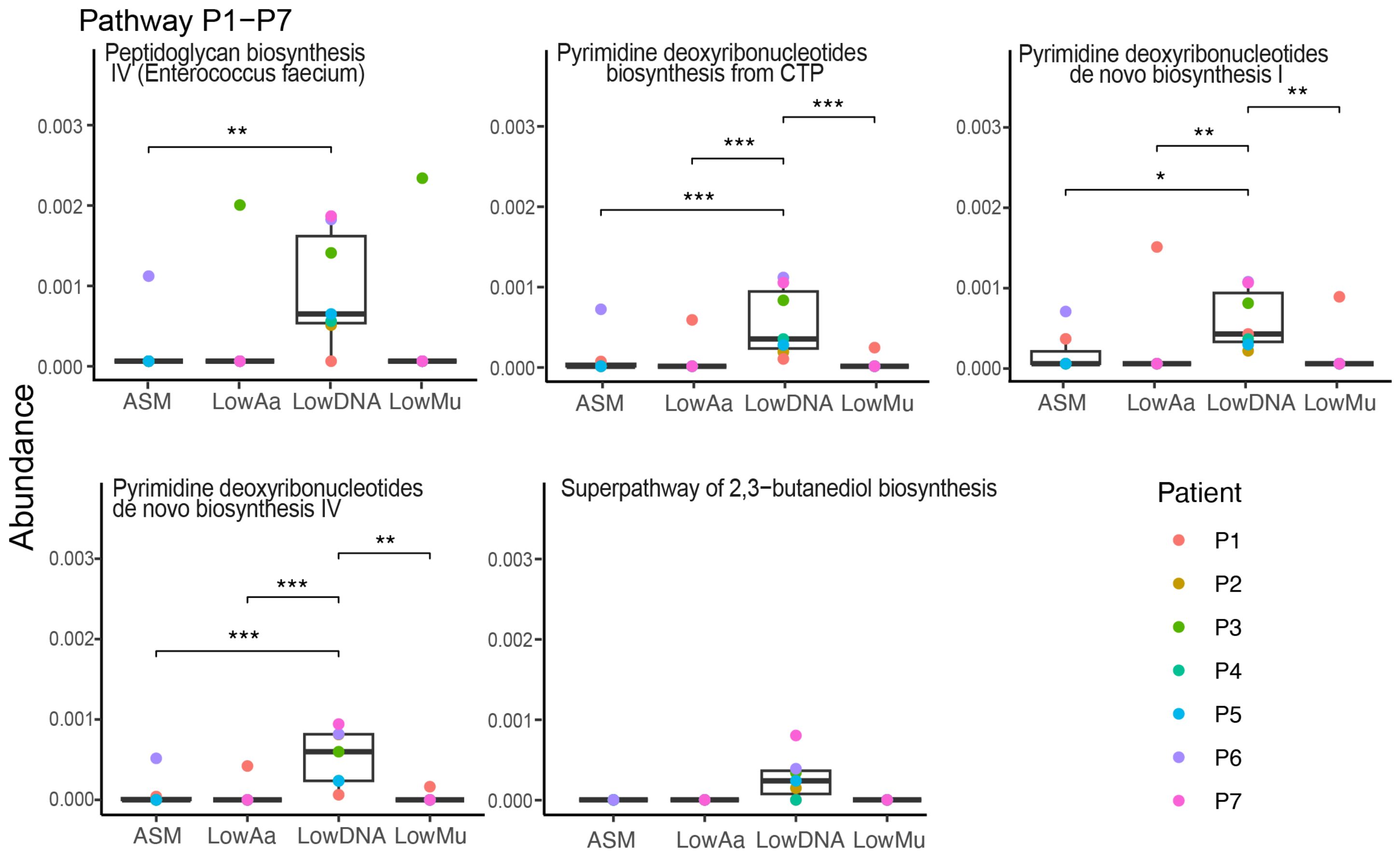

Figure S9. Metagenomic pathway relative abundance from aerobic sputum community P1 (*P. aeruginosa* dominates)-P7 data across the ASM and ndASM conditions.

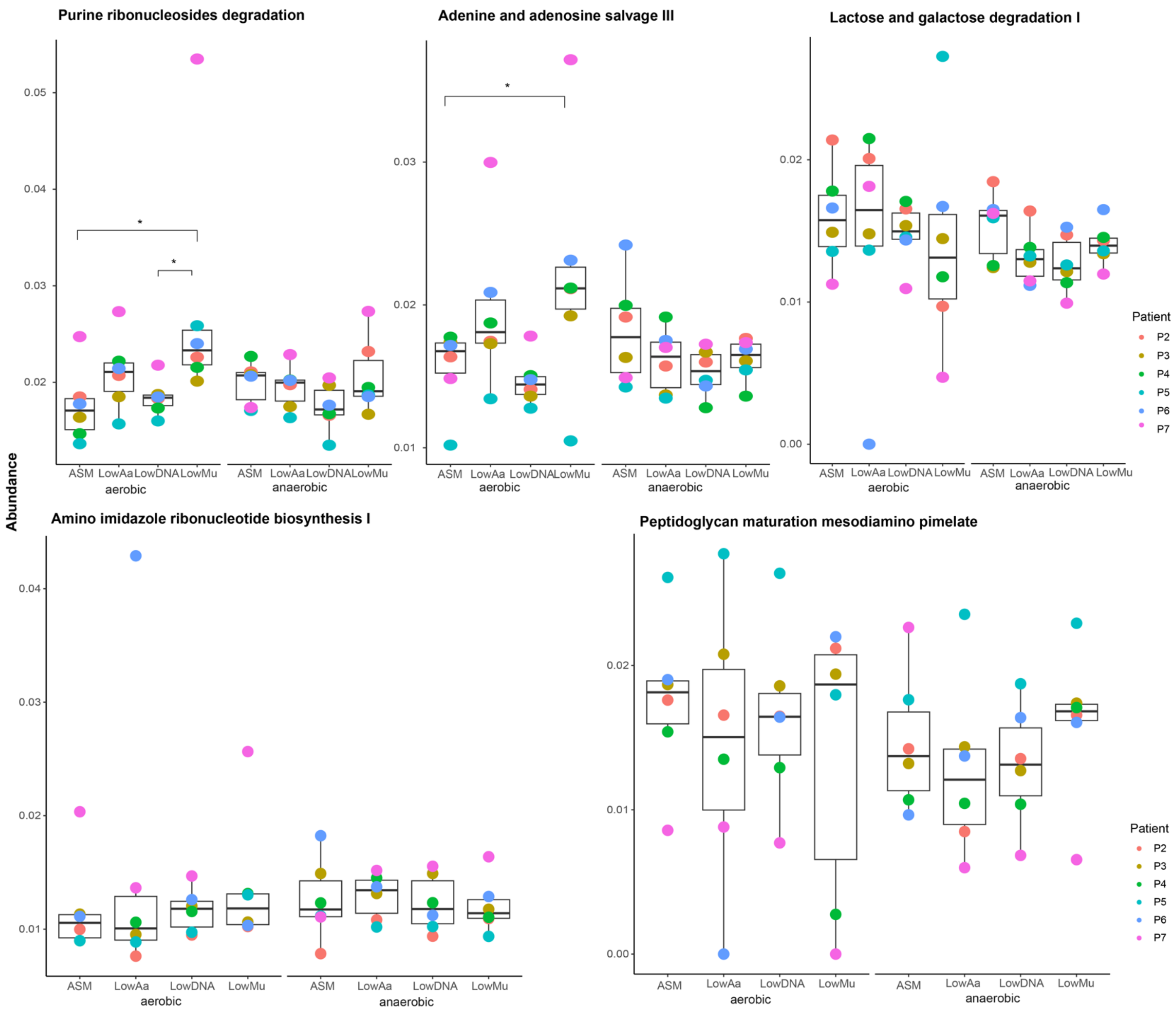

Figure S10. Metagenomic pathway relative abundance from aerobic and anaerobic sputum community P2-P7 data across the ASM and ndASM conditions.

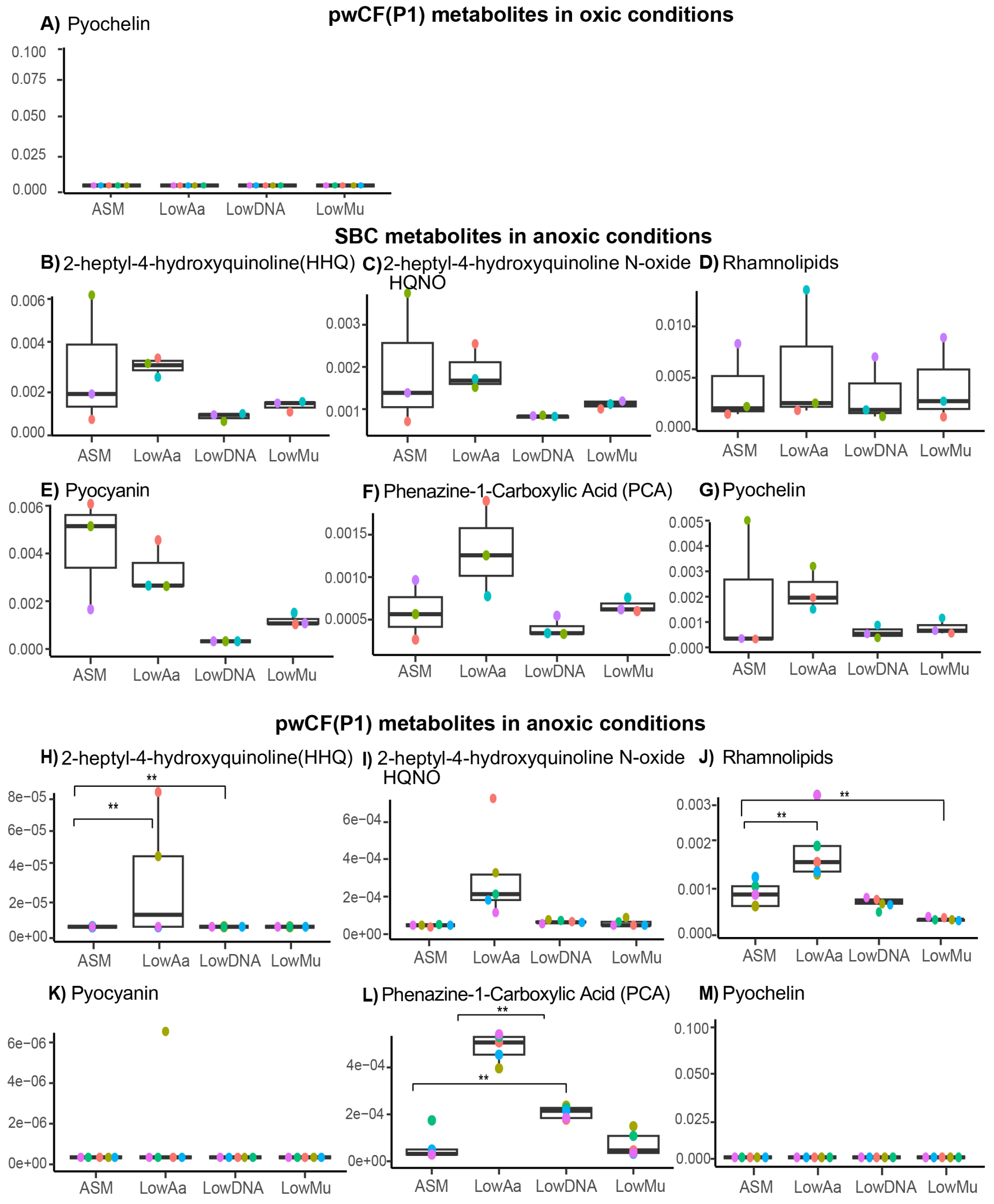

Figure S11. Quantitative analysis of metabolite production by *Pseudomonas aeruginosa* under different nutrient conditions. For plot a) Pyochelin was not produced in pwCF: P1 in aerobic conditions. Each plot (b-m) shows the concentration of specific metabolites or virulence factors measured under anaerobic conditions released by SBC. (a) HHQ (2-heptyl-4-hydroxyquinoline) levels, (b) HQNO (2-heptyl-3-hydroxy-4(1H)-quinolone N-oxide) levels, (c) Rhamnolipids levels, (d) Pyocyanin levels, (e) PCA (Phenazine-1-carboxylic acid) levels, (f) Pyochelin levels. pwCF (patient with cystic fibrosis) community P1 under anaerobic conditions, (g) HHQ, (h) HQNO, (i) Rhamnolipids, (j) Pyocyanin, (k) Pyochelin, (l) PCA, (m) Pyochelin. The graphs display mean values with error bars representing the standard deviation (SD) of three biological replicates.
